# Geometric regulation of histone state directs melanoma reprogramming

**DOI:** 10.1038/s42003-020-1067-1

**Published:** 2020-07-03

**Authors:** Junmin Lee, Thomas G. Molley, Christopher H. Seward, Amr A. Abdeen, Huimin Zhang, Xiaochun Wang, Hetvi Gandhi, Jia-Lin Yang, Katharina Gaus, Kristopher A. Kilian

**Affiliations:** 1 Department of Materials Science and Engineering, University of Illinois at Urbana-Champaign, Urbana, IL 61801 USA; 2 Institute for Genomic Biology, University of Illinois at Urbana-Champaign, Urbana, IL 61801 USA; 3 School of Chemistry, School of Materials Science and Engineering, Australian Centre for NanoMedicine, University of New South Wales, Sydney, NSW 2052 Australia; 4 Department of Cell and Developmental Biology, University of Illinois at Urbana-Champaign, Urbana, IL 61801 USA; 5 Prince of Wales Clinical School, University of New South Wales, Sydney, NSW 2052 Australia; 6 European Molecular Biology Laboratory Australia Node in Single Molecule Science and ARC Centre of Excellence in Advanced Molecular Imaging, School of Medical Sciences, University of New South Wales, Sydney, NSW 2052 Australia; 7 Department of Bioengineering, University of Illinois at Urbana-Champaign, Urbana, IL 61801 USA

**Keywords:** Biophysics, Cancer, Stem cells, Diseases

## Abstract

Malignant melanoma displays a high degree of cellular plasticity during disease progression. Signals in the tumor microenvironment are believed to influence melanoma plasticity through changes in the epigenetic state to guide dynamic differentiation and de-differentiation. Here we uncover a relationship between geometric features at perimeter regions of melanoma aggregates, and reprogramming to a stem cell-like state through histone marks H3K4Me2 and H3K9Ac. Using an in vitro tumor microengineering approach, we find spatial enrichment of these histone modifications with concurrent expression of stemness markers. The epigenetic modifier *PRDM14* overlaps with H3K9Ac and shows elevated expression in cells along regions of perimeter curvature. siRNA knockdown of *PRDM14* abolishes the MIC phenotype suggesting a role in regulating melanoma heterogeneity. Our results suggest mechanotransduction at the periphery of melanoma aggregates may orchestrate the activity of epigenetic modifiers to regulate histone state, cellular plasticity, and tumorigenicity.

## Abstract (web summary)

Junmin Lee et al. study the role of geometric features at the perimeter regions of melanoma aggregates in programming stem cell-like state through histone marks. They use a tumor microengineering approach in vitro and report a spatial enrichment of histone modifications with stemness markers. Their work uncovers a mechanotransduction signaling that regulates epigenetic modifiers to regulate tumorigenicity.

## Introduction

Malignant transformation and metastatic spread are known to be mediated by both genetic abnormalities^1^ and epigenetic alterations^2^. Epigenetics, defined as heritable change in gene expression occurring independent of changes in primary DNA sequence, is strongly implicated in the underlying mechanisms of cancer progression^3^. Microenvironment-mediated epigenetic regulation of cancer-related gene expression through DNA methylation, histone modification, and chromatin compartments is now believed to take part in a broad spectrum of cancer behaviors ranging from initiation to phenotypic alteration^4^. Histone modifications, including methylation and acetylation are covalent post-translational modifications to histone proteins. These modifications allow histones to alter the structure of chromatin, resulting in transcriptional activation or repression, that affect changes in cell behavior. For example, histone H3 lysine 4 di/tri-methylation (H3K4me2/3) and histone H3 acetylation (H3ac) are generally associated with gene activation^5^, whereas H3K27me, which marks active cis-regulatory elements, is associated with gene inactivation^6^. The detection of cancer-specific changes through histone modifications as epigenetic biomarkers has potential for clinical prediction, diagnosis, and therapeutic development.

Malignant melanoma-initiating cells (MICs), also referred to as melanoma repopulating cells or melanoma stem cells, are a dynamic sub-populations of cells that may arise during progression with tumor initiating capacities^7^. Unlike the clonal evolution model describing how a single cell accumulates genetic and epigenetic changes until becoming a cancer tumor cell^1^, the cancer stem cell model suggests a hierarchical organization (unidirectional) of cancer cells, according to their tumorigenic potential that has important implications for cancer therapy with stem cell-specific treatment regimens^8^. However, accumulating evidence surrounding cancer plasticity supports a new emerging model of tumorigenicity, in which dynamic plasticity facilitates malignant cells to revert to a stem cell-like phenotype^9^. Recently, we and other groups have shown that cancer cells are more plastic than previously anticipated. For instance, conversion to a stem cell-like state has been guided by microenvironment-mediated epigenetic regulation of gene expression including factors such as pH^10^, radiation^11^, stiffness^12^, hypoxia^13^, and interfacial stress^14^. These microenvironment parameters are not mutually exclusive and likely integrate in a context-dependent fashion during progression to guide tumor heterogeneity underlying progression. Hence, we hypothesize that if tumor cells are put into a specific context which facilitates reprogramming to the MIC phenotype, specific histone modifications may be used to understand the mechanisms underlying phenotypic alterations during progression. Our use of microengineering based on soft lithography allows us to mimic aspects of the tumor microenvironment, thus effectively deconstructing the biophysical cues of stiffness and geometry to probe how these parameters provide a context to facilitate epigenetic reprogramming to a stem cell-like tumorigenic state.

## Results

### Geometric cues regulate histone methylation and acetylation in melanoma

To classify histones linked to epigenetic reprogramming from melanoma to the MIC state, we employed microengineered hydrogels that we previously demonstrated will coordinate enhancement of the MIC phenotype with spatial control (Supplementary Fig. 1). In our previous study, B16F0 murine melanoma cells cultured for five days expressed higher levels of MIC markers at the periphery of microaggregates, with stem cell-like characteristics demonstrated in vitro and in vivo^14^. To understand how geometric cues at the perimeter of microaggregates of melanoma cells will influence histone state, we characterized a panel of histone marks that are implicated in controlling oncogenic gene activation. We first analyzed methylation state at Histone H3; H3K4me1/2/3 were studied because these are known as active histone marks^5^. In addition, Jarid1B (gene name: *KDM5B*) was also investigated because it is a common molecular marker of MICs, and is the histone lysine demethylase for H3K4me1/2/3 with pronounced roles in different cancer types^15^. For example, overexpression of Jarid1B in the MDA-MB 231 breast cancer cells suppressed malignant characteristics such as cell migration and invasion^16^, while overexpression of Jarid1B in melanoma^17^ or immortalized normal breast cancer cells (MCF10A)^18^ was found to enhance metastatic progression or cell invasion, respectively. Another representative histone mark associated with transcriptional activation, histone H3 lysine 36 di-methylation (H3K36me2), and a histone mark correlated with transcriptional repression, histone H3 lysine 9 tri-methylation (H3K9me3), were also employed.

**Fig. 1:**
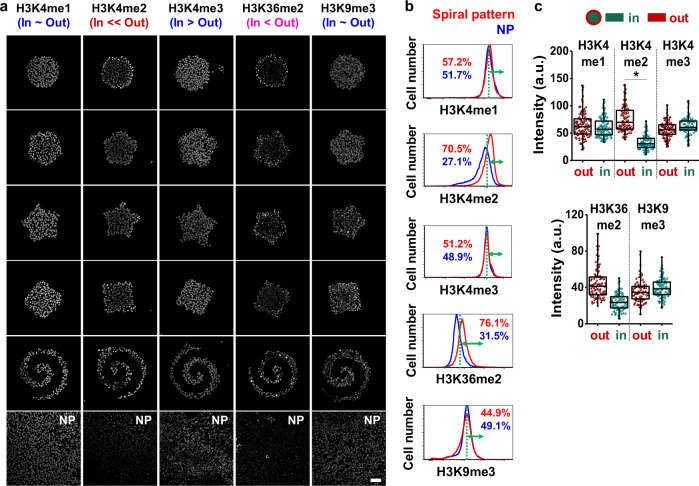
Histone methylation state is influenced by perimeter curvature. **a** Representative immunofluorescence images of H3K4me1/2/3, H3K36me2, and H3K9me3 for B16F0 cells cultured in a panel of shapes. **b** Flow cytometry characterization of histone methylation in B16F0 cells and quantification of the difference between patterned and non-patterned cells for each condition with thresholds (green dots) assigned by overlapping regions of patterned and non-patterned plots. **c** Single cell analysis of histone methylation in B16F0 cells cultured in perimeter or central regions of the circular geometry (*n* = 3, biological replicates, total 100 cells for each marker). Boxes represent 25^th^ to 75^th^ percentile and whiskers represent minimum-maximum. Horizontal lines and points within boxes represent the median and mean respectively for three duplicates. Scale bars, 50 μm. *P < 0.05, ANOVA. Error bars represent s.d.

We cultured cells for five days on five different micropatterned hydrogel substrates with the same area (50,000 μm^2^) or on non-patterned protein-stamped hydrogel substrates (10 kPa gels for both patterned and non-patterned) and immunostained for histone methylation state (Fig. 1a; Supplementary Fig. 2a, b). Interestingly, H3K4me2 and H3K36me2 expression co-localized with cells adopting the MIC phenotype at the periphery of microaggregates. We selected cells cultured for five days in the spiral shape for flow cytometry analysis because we previously found that this shape will augment the MIC phenotype^19^ through high interfacial boundary (perimeter/area) and high curvature^14^ (Supplementary Fig. 3). Similar to the immunofluorescence results, cells cultured in the spiral patterns display higher levels of H3K4me2 and H3K36me2 expression compared to those cultured on non-patterned surfaces (Fig. 1b; Supplementary Fig. 2c). To gain an understanding into the mechanism underlying the observed spatial distribution of histone marks, cells were grown in circular shapes, followed by quantification of histone marks in two different regions (outside and inside) within the same area. For the quentification, we employed the circular shapes in order to easily discern differences between two different regions (outside and inside) within the same area. Cells cultured at the perimeter displayed significantly higher levels of H3K4me2 compared to those cultured at central regions (Fig. 1c; Supplementary Fig. 2d), suggesting that regulation of gene expression associated with reprogramming of melanoma cells into the MIC phenotype could be linked to the H3K4me2 mark.

**Fig. 2:**
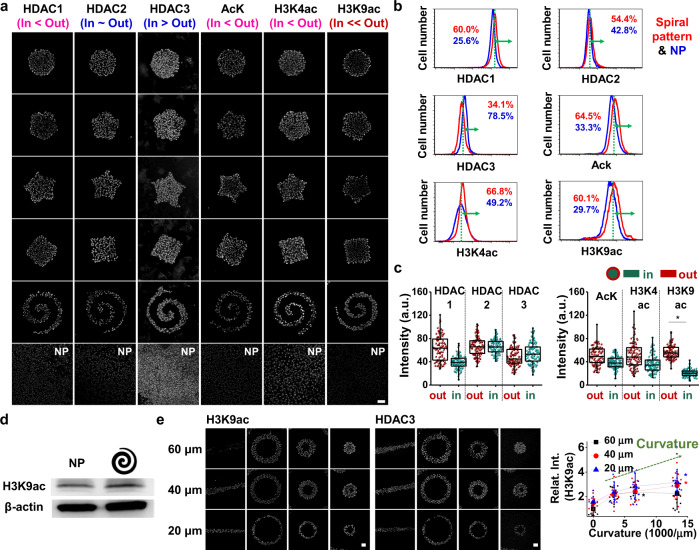
Histone acetylation and deacetylation correspond to epigenetic-mediated phenotype changes in melanoma. **a** Representative immunofluorescence images of histone acetylation and deacetylation for B16F0 cells cultured on a panel of shapes. **b** Flow cytometry characterization of histone acetylation and deacetylation in B16F0 cells and quantification of the difference between patterned and non-patterned cells for each condition with thresholds (green dots) assigned by overlapping regions of patterned and non-patterned plots. **c** Single cell analysis of histone acetylation and deacetylation in B16F0 cells cultured in two different regions of the circular shape (*n* = 3, biological replicates, total 100 cells for each marker). Boxes represent 25^th^ to 75^th^ percentile and whiskers represent minimum-maximum. Horizontal lines and points within boxes represent the median and mean respectively for three duplicates. **d** Western blots for H3K9ac with non-patterned or spiral-patterned melanoma cells. **e** Shapes regulating curvature and perimeter/area to explore the relationship between H3K9ac and HDAC3 (*n* = 3, biological replicates, total 12 patterns for each condition, except for 20 μm-width line shape: 7 patterns and 40 μm-width medium torus shape: 9 patterns). Significant differences were compared to those cultured on 60 μm line shapes. Scale bars, 50 μm. *P < 0.05, ANOVA. Error bars represent s.d.

Histone acetylation is also an important modification in regulating chromatin accessibility and regulation of gene expression^20^, and various histone acetylation states have been shown to control gene expression during cancer progression^21^. To probe acetylation activity in our micropatterned cultures, we selected a panel of class I histone deacetylases (HDAC) and measured global acetylation of lysine (AcK), and histone H3 lysine 4 and 9 (H3K4ac and H3K9ac) marks which are associated with gene activation. By applying the same process for identifying methylation states involved in perimeter activation of MICs, we found that cells cultured at the periphery of different shapes expressed higher levels of HDAC1, AcK, H3K4ac, H3K9ac, and lower levels of HDAC3 compared to those cultured at central regions (Fig. 2a; Supplementary Fig. 4a, b). Flow cytometry of cells cultured in spiral patterns or on non-patterned substrates supported these immunofluorescence results (Fig. 2b; Supplementary Fig. 4c). Regional analysis reveals that cells cultured at the periphery of shapes exhibit significant elevation of the H3K9ac mark (Fig. 2c, d; Supplementary Fig. 4d, e), corresponding to higher expression levels of MIC and stemness markers (Supplementary Fig. 5). AcK and histone marks H3K4ac showed perimeter enhancement, although the difference was not statistically significant. We also immunostained cells cultured along straight lines and ring shapes where curvature (the convex curvature at the exterior of the torus shapes) and perimeter/area ratio can be varied. After five days in culture, we see cells show higher levels of H3K9ac with increased perimeter curvature and P/A, with a modest reduction in HDAC3 expression, although the difference is not statistically significant. While preliminary, this result supports a potential role for HDAC3 in deacetylating H3K9ac (Fig. 2e; Supplementary Fig. 6).

To investigate the role of Jarid1B, a histone demethylase for H3K4me1/2/3 and an established molecular marker for MICs^15^ in demethylase activity associated with the stem cell-like state, we cultured B16F0 cells in spiral geometries with small interfering RNA (siRNA) of Jarid1B with scrambled siRNA as control. siRNA concentration and delivery time were adjusted to ensure cells in control and experimental conditions reached approximately the same confluence. After five days in culture, we performed gene expression analysis using quantitative polymerase chain reaction (qPCR) of a panel of markers associated with the MIC state (*CD271*, *SOX2*, *OCT4*, and *Nanog*). We acknowledge that these markers and the existence of the MIC state are controversial; however these markers are accepted regulators of stemness phenotypes. We see a lower degree of transcript expression for markers associated with stemness for cells cultured with Jarid1B siRNA, except for transcript expression of *CD271* at the siRNA concentration of 25 nM (Supplementary Fig. 7a–f). To evaluate the potential role of Jarid1B in regulating the H3K4me1/2/3 histone marks across spatial regions, we performed immunofluorescence staining of H3K4me1/2/3 for cells cultured in circular shapes, treated with Jarid1B or scrambled siRNA. Jarid1b knockdown does not change the levels of H3K4me2, while leading to an increase in the H3K4me3. This suggests Jarid1b is involved in demethylation of H3K4 but not necessarily associated with regulation of the MIC state at geometric features (Supplementary Fig. 7g–i). Interestingly, we also see a lower degree of transcript expression of HDAC1 for cells cultured with Jarid1B siRNA (Supplementary Fig. 7e), this may be because HDAC1 is one of the nucleosome remodeling and deacetylase (*NuRD*) complex which physically and functionally interacts with Jarid1B for transcriptional remodeling^15^ and one of the EMT-inducing genes (*Snail*) when complexed with HDAC2^22^.

### Histone 3 lysine 4 di-methylation and lysine 9 acetylation regulates oncogenic gene expression

**Fig. 3:**
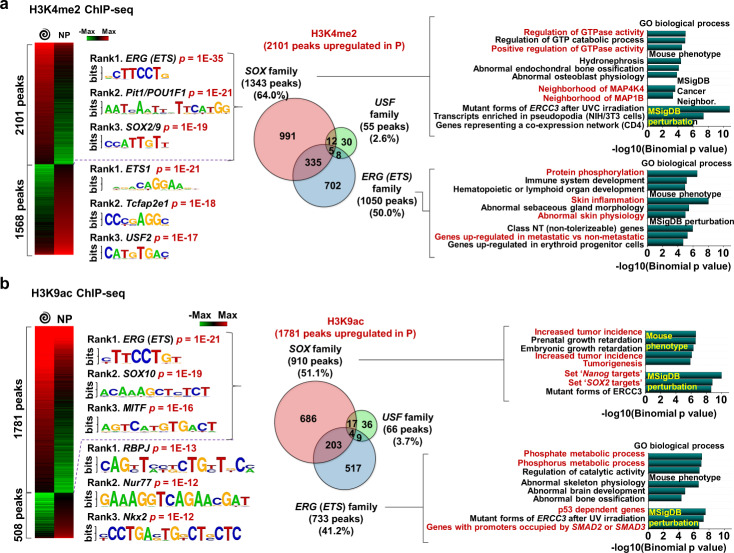
H3K4me2/H3K9ac-regulated gene panels predict phenotypic alterations of melanoma. Heatmap of **a** H3K4me2 and **b** H3K9ac ChiP-seq results for cells cultured on spiral geometry or non-patterned substrates. The top three high ranked regulatory motifs cut by *p*-values. Venn diagram showing the number of enriched genes for cells cultured on spiral patterns linked to *SOX*, *ETS*, and *USF* families among H3K4me2-marked genes.

To understand the possible mechanisms underlying changes in cell state on account of specific histone marks, B16 melanoma cells were grown on spiral patterned (reprogramming) or non-patterned (control) hydrogel substrates for five days, followed by chromatin immunoprecipitation and DNA sequencing (ChIP-seq). ChIP assays specific for the identified histone marks (H3K4me2/H3K9ac) were performed and differential ChIP peaks were identified with at least a 2-fold change and 0.001 FDR cutoff (Fig. 3a, b). More differential H3K4me2 (57.3%)/H3K9ac (77.8%) peaks were shown for the cells cultured in spiral geometries. To gain insights into potential regulators at these differential sites such as DNA-binding transcription factors, we also performed motif enrichment analysis. We found that differential peaks in spiral patterned versus non-pattered cells are enriched for distinct motif families; *ERG (ETS)*/*Pit1*/*SOX2/9* (reprogramming) or *ETS1*/*TcFap2e1*/*USF2* (control) for H3K4me2 differential peaks and *ERG (ETS)*/*SOX10*/*MITF* (reprogramming) or *RBPJ*/*Nur77*/*Nkx2* (control) for H3K9ac differential peaks. *ETS* genes are known to be linked to p38/ERK mitogen-activated protein kinases (MAPK) signaling for tumor growth and progression^23^. For example, *ETS1* could promote the development and invasion of malignant melanoma^24^, and when it associated with *RhoC* (also enriched for cells on spiral patterned hydrogels), melanoma cells could be progressive and metastatic^25^. Although the *ETS* family was also a top ranked motif for H3K4me2 peaks in non-patterned cells, enriched gene annotation associated with the differential peaks (Supplementary Figs. 8 and 9a) suggest a distinct role in coordinating the MIC phenotype. *Pit1* is also known to upregulate *Snai1*, leading to tumor epithelial-mesenchymal transition (EMT) and their growth and metastasis^26^. Similar trends were observed for H3K9ac peaks but it has more distinct and specific differences between cells cultured on spiral patterned and non-patterned hydrogel substrates (Fig. 3b). We acknowledge that our data show a correlation between the geometry and the histone marks but may not indicate a causative role. First, we found over 50 predictive transcription factor motifs associated with H3K9ac differential peaks for patterned cells over non-patterned cells (top 16 predictive transcription factor motifs, Supplementary Fig. 9b). Among them we selected the top 3 peaks based on *P*-values—*ERG(ETS)*, *SOX10*, and *MITF*.

### Histone H3 lysine 9 acetylation influences MIC state through *SOX10* motif

**Fig. 4:**
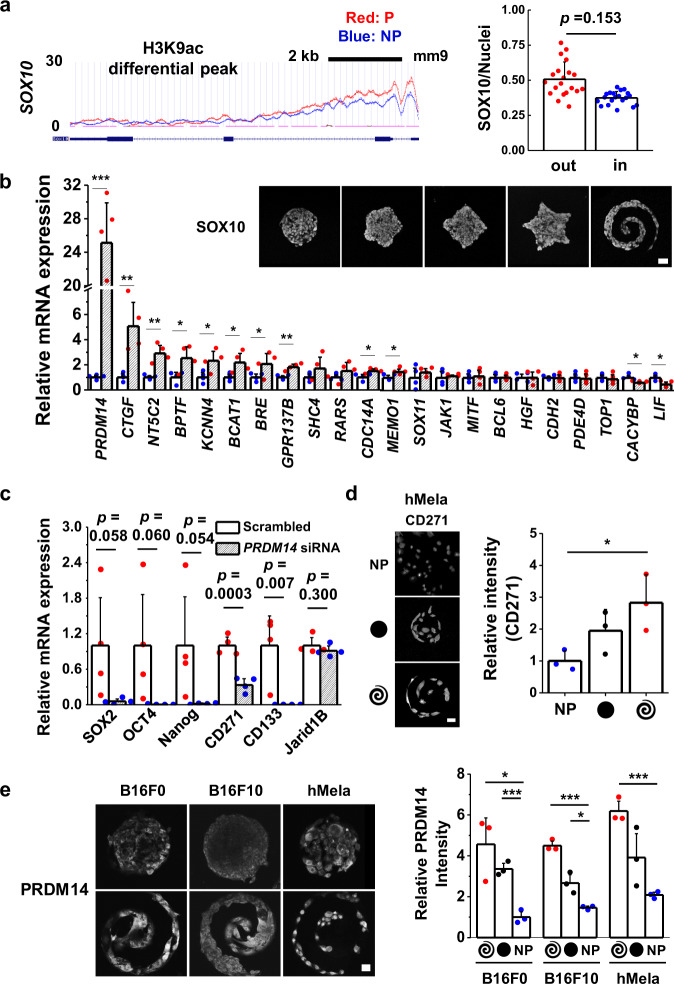
*SOX10* and *PRDM14* are involved in regulating the epigenetic state associated with the MIC phenotype. **a** ChiP-seq occupancy for H3K9ac over *SOX10*, intensity ratio of SOX10 normalized by the fluorescent signal to the intensity of DAPI (*n* = 3, biological replicates, total 20 patterns for each condition), and representative immunofluorescence confocal images of SOX10 for B16F0 cells cultured in a panel of shapes. **b** Results of qPCR to measure the expression of genes associated with the differential peaks of H3K9ac/*SOX10* motif. (*n* = 4, biological replicates) **c** Results of qPCR to measure the gene expression of stemness (*SOX2*, *OCT4*, *Nanog*) and MIC (*CD271*, *CD133*, *Jarid1B*) for B16F0 cells cultured on spiral geometries for 5 days with *PRDM14* or scrambled siRNAs (*n* = 4, biological replicates). **d** Representative immunofluorescence images and relative intensity of representative MIC marker, CD271 for hMela cells cultured for five days on circular or spiral geometries or non-patterned substrates. (*n* = 3, biological replicates, total 12 patterns for each condition). **e** Representative immunofluorescence confocal image and relative intensity of PRDM14 expression for B16F0, B16F10, and hMela cells cultured for five days on circular or spiral geometries or non-patterned substrates. (*n* = 3, biological replicates, total 12 patterns for each condition). Scale bars, 50 μm. *P < 0.05, **P < 0.005, ***P < 0.0005, ANOVA. Error bars represent s.d.

One of the top motifs associated with H3K9ac for cells cultured in regions of high curvature and perimeter/area is *SOX10*, a neural crest stem cell marker. Previous studies revealed that *SOX10* played an important role in melanoma cell survival, proliferation, and metastasis^27^. It was also reported that the CD271 expression for malanoma, one of representative markers for the MIC state, was directly related to the expression of *SOX10*^28^. In addition, previous studies showed that *MITF* which could function as a melanoma oncogene was associated with melanoma progression^29^, and *SOX10* is known to act upstream of *MITF*^30^, meaning that *SOX10* may thus contribute to the melanoma-specific expression of genes associated with the MIC state. Interestingly, the enriched mouse phenotype annotations related to *SOX10* family in H3K9ac peaks for reprogrammed cells suggest that increased tumor incidence and tumorigenesis are involved in their mouse phenotype. Furthermore, *Nanog* and *SOX2* targets may be perturbed by the *SOX10* family, suggesting the importance of *SOX10* in activation of cells to the MIC state at the tumor periphery. Since we found that the *SOX10* motif was enriched inside the differential histone peaks (Fig. 3b), we conducted immunofluorescence for *SOX10* on our microaggreagates as well as ChIP-seq of *SOX10*. Cells cultured at the perimeter of microaggregates express higher levels of *SOX10* compared to those cultured in central regions (Fig. 4a; Supplementary Fig. 10), and we see 14 differential peaks associated with cells cultured on patterned gels compared to those cultured on non-patterned gels. Some genes like *Med27* and *Trim14* inside H3K4me2 peaks were shown as one of the best differential *SOX10* peaks associated with activated cells, and some peaks located nearby *Klf12*, *Scml4*, and *Dync1li2* were intersected with differential H3K9 peaks (Supplementary Fig. 11). These genes may also be involved in malignant melanoma transformation. In addition, *SOX10* appears to be binding a number of interesting genes in B16 melanoma as shown in the top enriched GO category regulating transcription as well as the other TFs that *SOX10* binds nearby in cells, which could be playing a role in the transition (Supplementary Fig. 12).

### Melanoma initiating cell phenotypes at the perimeter are directed by PR/SET domain-containing 14 (*PRDM14*)

To further confirm the association between the high ranked regulatory motifs (*ERG (ETS)*, *SOX10*, and *MITF* for activated cells upregulating H3K9ac peaks) and regulation of downstream target genes, we identified H3K9ac differential peaks between two different conditions (reprogrammed and control cells). We first looked at the list of H3K9ac differential peaks to narrow down genes associated with cancer growth and progression. Among them, a number of these differential peaks were located in the regulatory domains of genes associated with cancer growth and progression and thus, we analyzed the expression of these genes for reprogrammed cells over control. Cells were cultured for 5 days on patterned substrates followed by lysis, RNA isolation and qPCR. Interestingly, activated cells cultured on patterned substrates show significantly higher expression of genes related to malignant melanoma such as *CTGF* (~5-fold) and *NT5C2* (~3-fold) compared to cells cultured on non-patterned substrates (Fig. 4b). The most highly differentially expressed gene associated with H3K9ac/*SOX10* is *PRDM14* (~25-fold). *PRDM14* is an epigenetic modifier involved in regulating pluripotency of stem cells with a clear role in modulating expression of core transcription factors *Nanog*, *SOX2*, and *OCT4*^31–35^. *PRDM14* is required to repress genes associated with lineage commitment and ensures naïve pluripotency in embryonic stem cells^34^. In addition, *PRDM14* has been implicated in affecting the severity of several human cancers including breast^36^ and leukemia^37^ with evidence of a link to regulating a stem cell-like state^37^. To date *PRDM14* has not been linked to tumorigenicity and the MIC state in melanoma. These findings are supported by the number of overlaps for peak co-occurrence of *PRDM14* (pre-existing *PRDM14* ChiP-seq data in the literature)^38^ with the H3K9ac and H3K4me2 marks from our study within 1000 bp, showing significant overlap with all of the peak sets and differential peak sets, except for PvsNP_k4me2 (Supplementary Table 1).

To explore how *PRDM14* plays a supportive role in coordinating the epigenetic state of melanoma in response to perimeter geometry, we performed knockdowns of *PRDM14* using siRNA. Knockdown was verified by qPCR and Western analysis using three different siRNAs against *PRDM14* with a scrambled control (Supplementary Fig. 13a, b). *PRDM14* knockdown resulted in a decrease in expression for genes linked to the MIC state; knockdown of *PRDM14* led to complete abolishment of pluripotency markers *SOX2*, *OCT4*, and *Nanog*, and putative MIC markers *CD271* and *CD133*; *PRDM14* knockdown led to a partial decrease in *Jarid1B* expression (Fig. 4c), which was in line with the immunofluorescnence analysis displaying there may be a functional relation between *PRDM14*-mediated pluripotency and these specific histone modifications (Supplementary Fig. 13c). To confirm the localization of PRDM14 at perimeter features of micropatterned melanoma aggregates, we performed immunofluorescence characterization in both B16 cells and human primary melanoma cells. Fig. 4d shows perimeter enrichment of PRDM14 in the B16F0 cells and to a lesser extent the B16F10 cells. Both Melanoma B16F0 and B16F10 were all derived from C57B1/6 mice; the B16F0 model remains close to the parental B16F1 and grows slowly, whereas the B16F10 model is very aggressive, much more than the B16F0 model. The higher localization at the periphery in the B16F0 cells is consistent with a mechanism where *PRDM14* is activated at the interface to reprogram cells of low metastatic potential to a highly metastatic MIC phenotype. To verify our observations in human cells, we chose to look at a primary human melanoma cell line (hMela). Human melanoma cells show significant enhancement in both CD271 and PRDM14 (Fig. 4d, e; Supplementary Fig. 13d), suggesting this mechanism is not unique to mouse cell lines but may play a role in guiding the MIC phenotype in human cells.

## Discussion

In this paper we demonstrate how changes in specific histone marks at the perimeter of melanoma aggregates correspond to phenotypic alteration of melanoma cells to a stem cell-like MIC state. We show that stress exerted on microconfined cells at the periphery primes the melanoma phenotype through epigenetic reprogramming via histone modifications H3K9ac and H3K4me2, and involvement of the epigenetic modifier *PRDM14*. While we have shown how geometric cues can give rise to differential activity of *PRDM14*, it remains to be demonstrated how *PRDM14* is activated based on these biophysical inputs. Furthermore, there are many other histone marks that remain to be profiled in order to map the complexity of epigenetic reprogramming in melanoma. For instance, a recent study demonstrated a link between H3K27me3 and mouse germ cell migration^39^. Nevertheless, our ChIP-seq analysis serves to illuminate how mechanotransduction can regulate the activity of stemness-related epigenetic modifiers including *PRDM14* (Fig. 4c).

The mechanistic basis of geometry-driven epigenetic changes may be related to the response of tumors to their microenvironment, where intratumor pressure, extracellular mechanics, and curvature at the margin coordinate to provide a context in which mechanotransduction and downstream gene expression are regulated by these multivariate signals. Since these microenvironment parameters coincide with tumor angiogenesis^19^, it is important to consider the ramifications of transformation to a MIC state in proximity to pathways for metastasis. These findings may help guide researchers in further exploring epigenetic signatures for tumor malignancy, and the development of strategies to prevent, diagnose, and treat metastatic cancers.

## Methods

### Hydrogel fabrication

10 kPa polyacrylamide hydrogels (PA) were made as described previously^14^. Briefly, 10% acrylamide and 0.1% bis-acrylamide (Sigma) solution were prepared and mixed with initiators, 0.01% ammonium persulfate (APS, Sigma) and tetramethylethylenediamine (TEMED, Sigma), to initiate gelation. 20 µl of the mixture was sandwiched between a glass coverslip (18 mm, Fisher Scientific) functionalized with 3-aminopropyltriethoxysilane for 3 min and glutaraldehyde for 30 min (Sigma) and a hydrophobically treated glass slide to generate the even and homogeneous surface of gels on the activated coverslips. After around 25 min of gelation, the coverslips conjugated with gels were gently detached from the hydrophobically treated glass slide. Hydrazine hydrate (55%) chemistry was employed to modify the surface chemistry of PA gels and applied to the gels surface for 2 h with rocking, followed by washing with 5% glacial acetic acid for 1 h and distilled water for 1 h. PA gels fabricated on coverslips were stored at 4 °C for later use. Polydimethylsiloxane (PDMS, Polysciences) stamps were fabricated from silicon masters made by conventional photolithography for patterned or non-patterned shapes. To generate free aldehydes from oxidize sugar groups in matrix protein (fibronectin, Sigma), sodium periodate (~3.5 mg/ml, Sigma) for at least 45 min was employed. The protein solution was mounted onto patterned or non-patterned (flat surface) stamps for 30 min and dried with air. Micro-contact printing was used to transfer the protein residues on stamps to the gel surface (chemical conjugation).

### Cell source and culture

The cancer cell lines B16F0 and B16F10 cells were purchased from American Type Culture Collection (ATCC) and cultured according to the recommended protocols. Media was changed every 3 to 4 days and cells were passaged at nearly 90% confluence using 0.25% trypsin (please see Supplementary Table 2 for reagent information). B16F0 cells were verified for mycoplasma contamination at Charles River Laboratories for cell line testing. De-identified human melanoma cell lines were a kind gift from John A. Copland III from the Mayo Clinic, Jacksonville, FL.

### Immunofluorescence

B16F0, B16F10, or hMela cells were fixed using 4% paraformaldehyde for 20 min. Cells were permeabilized with 0.1% Triton X-100 for 30 min at room temperature and then blocked with 1% bovine serum for 15 min. Cells were stained with the appropriate primary and secondary antibodies (Supplementary Table 3). Before every step, cells were washed at least twice with PBS. Imaging was performed using an LSM 700 (Carl Zeiss, Inc.) four laser point scanning confocal microscope with a single pinhole for confocal imaging for fluorescence imaging.

### RNA isolation and qPCR

Adherent cells on patterned gels (12 identical substrates for each condition) were lysed directly in TRIZOL reagent (Invitrogen). Total RNA was isolated by chloroform extraction and ethanol precipitation and amplified using TargetAmp™ 1-Round aRNA Amplification Kit 103 (Epicenter) according to vendor protocols. Superscript III® First Strand Synthesis System for qPCR (Invitrogen) was employed to reversely transcribe total RNA. qPCR was performed using SYBR® Green qPCR Master Mix (Invitrogen) on an Eppendorf Realplex 4S qPCR system. Primer sequences were in supplementary Table 4. All reactions were performed linearly by cycle number for each set of primers.

### Western analysis

Cell extracts were isolated using RIPA buffer supplemented with protease and phosphatase inhibitors. Protein concentrations were determined by Nanodrop or BCA protein assay (Thermo Fisher Scientific), according to the company instructions. Subsequently, proteins were separated by 4–20% SDS-PAGE and electrophoretically transferred onto PVDF (Thermo Fisher Scientific) or nitrocellulose membranes (Bio-rad, Australia), which were then probed with primary antibodies overnight at 4 °C. HRP-conjugated secondary antibodies were detected by chemiluminescence agent ECL or Supersignal Western Dura Extended Duration (Thermo Fisher Scientific, Australia). Membranes were imaged by ImageQuant LAS4000 (GE healthcare, Sweden). Densitometric analysis was performed by ImageQuant TL Software (GE healthcare) and presented as ratios of protein expression normalized to relevant *GAPDH* or *β-actin* loading control.

### RNA Interference

The siRNAs for *Jarid1B* (ID 75605, Trilencer-27 Mouse siRNA, siRNA A: SR422988A, siRNA B: SR422988B, and siRNA C: SR422988C) or scrambled siRNAs (SR30004) as well as *PRDM14* (ID  383491, Trilencer-27 Mouse siRNA, siRNA A: SR417406A, siRNA B: SR417406B, and siRNA C: SR417406C) or scrambled (SR30004) were purchased from OriGene. Transfection was performed according to the vendor’s instructions. Lipofectamine 2000™ was employed for higher transfection efficiency. Cells cultured for 5 days in patterned substrates were treated with siRNA twice at day 1 and day 3.

### Cell labeling and flow cytometry

B16F0 cells cultured for five days on spiral-patterned or non-patterned gels (12 identical substrates for each condition) were isolated from substrates by trypsin, followed by breaking down into a single cell suspension. Cells were fixed in 4% paraformaldehyde for 20 min and then permeabilized in 0.1% Triton X-100 in PBS for 30 min. After blocking cells in 1% BSA in PBS for 1 h, Cells were stained with primary antibodies in 1% BSA in PBS overnight at 4 °C and then secondary antibodies in 2% goat serum, 1% BSA in PBS for 20 min in a humid chamber (5% CO_2_ and 37 °C). Before every step, cells were rinsed at least three times with PBS. A BD LSR Fortessa Flow Cytometry Analyzer was used to perform flow cytometry analysis. To set the baseline, negative controls were prepared by staining cells without primary antibodies.

### Microscopy data analysis

Confocal images were analyzed using ImageJ software. Multiple cells were imaged for each condition and fluorescence intensities of single cells in different regions of patterns (after background subtraction) were used to compare marker expression. For generating immunofluorescence heatmaps, cells cultured on various shapes were fixed, stained, and imaged on the same day using the same settings. After subtraction of background intensities of raw fluorescent images, patterns were aligned in ImageJ with the same orientation as cultured across the surface, followed by incorporating into a Z stack with the average intensity calculated for heatmap generation.

### Chromatin immunoprecipitation and sequencing (Chip-seq)

H3K4me2 and H3K9ac ChiP samples were prepared from B16F0 cells cultured on patterned or non-patterned substrates, and ChiP DNA quality was verified as previously described^40^. B16 melanoma cells were cultured for five days and then fixed with 1% formaldehyde for 10 min at room temperature. Fixations were quenched by glycine (125 mM), followed by washing cells with cold 1x PBS two times. Cells were treated with hypotonic lysis buffer for 10 min (20 mM HEPES at pH 7.9, 10 mM KCl, 1 mM EDTA at pH 8, 10% glycerol, 1 mM DTT, 0.5 mM PMSF, 0.1 mM sodium orthovanadate, and 1X Roche protease inhibitors). Collected nuclear pellets were lysed in in 1× RIPA buffer (10 mM Tris-Cl at pH 8.0, 140 mM NaCl, 1% Triton X-100, 0.1% SDS, 1% deoxycholic acid, 0.5 mM PMSF, 1 mM DTT, 0.1 mM sodium orthovanadate, and Roche protease inhibitors). Nuclear lysates were sonicated with a Branson 250 Sonifier (output 20%, 100% duty cycle) to shear the chromatin to ∼1 Kb in size. Clarified lysates were incubated overnight at 4 °C with anti-H3K4me2 (Cell Signaling, 9725) or H3K9ac (Cell Signaling, 9649) antibodies. Protein–DNA complexes were precipitated, immunoprecipitates were washed three times in 1× RIPA, once in 1× PBS, and then eluted from the beads by addition of 1% SDS, 1× TE (10 mM Tris-Cl at pH 7.6, 1 mM EDTA at pH 8), and incubated for 10 min at 65 °C. Cross-links were reversed overnight at 65 °C. Purification for all samples were performed by treatment first with 200 μg/mL RNase A for 1 h at 37 °C, then with 200 μg/mL Proteinase K for 2 h at 45 °C, followed by extraction with phenol:chloroform:isoamyl alcohol and precipitation at −70 °C with 0.1 volume of 3 M sodium acetate, 2 volumes of 100% ethanol, and 1.5 μL of pellet paint coprecipitant. ChIP DNA prepared from 1 × 10^7^ cells was resuspended in 50 μL of ultrapure water. Sequencing (an Illumina HiSeq 2500 sequencer using a TruSeq SBS sequencing kit, version 4) was performed and Fastq files were obtained and demultiplexed with the bcl2fastq v2.17.1.14 Conversion Software (Illumina). The sequencing data from this study have been submitted to the NCBI Gene Expression Omnibus (GEO; http://www.ncbi.nlm.nih.gov/geo/).

### Chip-seq data analysis

ChIP-seq bioinformatics analyses were performed. Sequence data were mapped with Bowtie2 (Langmead and Salzberg 2012) to the UCSC Mus musculus mm9 genome, using default settings. Mapped sequence data were analyzed for peaks using HOMER (Hypergeometric Optimization of Motif EnRichment) v4.7 (Heinz et al. 2010). Samples were converted into tag directories, and QC was performed using read mapping and GC bias statistics. Histone peaks were then called from the Tag Directories with default factor settings, except local filtering was disabled (-L 0) and input filtering was set at three-fold over background (-F 3), to increase the sensitivity of the peak calling and identify individual subunits of multihistone peaks. After peak calling, peak files were annotated to the mouse mm9 genome using HOMER’s annotation script to assign peaks to genes, and associate peaks with differential expression data. BigWiggle pileup files were generated using HOMER’s makeBigWig.pl script with default settings. Differential chromatin peaks were identified using the HOMER getDifferentialPeak.pl script, looking for any peaks that changed at least two-fold between conditions with a significance cutoff of 1 × 10^–4^. Genes annotated nearby differential peaks were submitted for GO analysis to DAVID and GREAT. Motif Analysis was performed with the HOMER findMotifsGenome.pl script using default settings.

### Statistics and reproducibility

Data were obtained at least three independent experiments and error bars represent standard deviation around the mean unless otherwise specified. For comparing statistics between two groups or more than two groups, student’s *t*-test or analysis of variance (ANOVA) with Tukey HSD Post-hoc testing, respectively, were employed. Differences were considered significant at *P* < 0.05.

### Reporting summary

Further information on research design is available in the Nature Research Reporting Summary linked to this article.

## Supplementary information

Reporting Summary

Description of Additional Supplementary Files

Supplementary Data 1

Supplementary Information

## Data Availability

Genome-wide maps of chromatin state in mouse B16 melanoma cancer cells in response to interfacial geometry were submitted to the GEO accession numbers GSE146444. The authors declare that all data supporting the findings of this study are available within the article, its Supplementary Information file and from the corresponding author on reasonable request. The source data underlying the graphs presented in the main and supplementary figures are shown as Supplementary Data 1. The uncropped western blot images corresponding to Fig. 2d and Supplementary Fig. 13b are shown in Supplementary Figs. 14 and 15, respectively.
