# Supplementary material for: Geometric regulation of histone state directs melanoma reprogramming
Source: Commun Biol. 2020 Jul 3;3:341. doi: 10.1038/s42003-020-1067-1 (PMC7334222; doi:10.1038/s42003-020-1067-1)
Supplement: Supplementary file 1 — Reporting Summary [file 42003_2020_1067_MOESM1_ESM.pdf]

nature research

Corresponding author(s): Dr. Kristopher A. kilian

Last updated by author(s): Feb 24, 2020

Reporting Summary

Nature Research wishes to improve the reproducibility of the work that we publish. This form provides structure for consistency and transparency in reporting. For further information on Nature Research policies, see Authors & Referees and the Editorial Policy Checklist.

Statistics

For all statistical analyses, confirm that the following items are present in the figure legend, table legend, main text, or Methods section.

n/a	Confirmed	
☐	☒	The exact sample size (n) for each experimental group/condition, given as a discrete number and unit of measurement
☐	☒	A statement on whether measurements were taken from distinct samples or whether the same sample was measured repeatedly
☐	☒	The statistical test(s) used AND whether they are one- or two-sided<br>*Only common tests should be described solely by name; describe more complex techniques in the Methods section.*
☒	☐	A description of all covariates tested
☐	☒	A description of any assumptions or corrections, such as tests of normality and adjustment for multiple comparisons
☐	☒	A full description of the statistical parameters including central tendency (e.g. means) or other basic estimates (e.g. regression coefficient) AND variation (e.g. standard deviation) or associated estimates of uncertainty (e.g. confidence intervals)
☐	☒	For null hypothesis testing, the test statistic (e.g. F, t, r) with confidence intervals, effect sizes, degrees of freedom and P value noted<br>*Give P values as exact values whenever suitable.*
☒	☐	For Bayesian analysis, information on the choice of priors and Markov chain Monte Carlo settings
☒	☐	For hierarchical and complex designs, identification of the appropriate level for tests and full reporting of outcomes
☒	☐	Estimates of effect sizes (e.g. Cohen's d, Pearson's r), indicating how they were calculated

Our web collection on statistics for biologists contains articles on many of the points above.

Software and code

Policy information about availability of computer code

Data collection	Fastq files were obtained and demultiplexed with the bcl2fastq v2.17.1.14 Conversion Software (Illumina).
Data analysis	ImageQuant TL Software (GE healthcare) was used for densitometric analysis (western). ImageJ software was used for analysis of confocal images. Sequence data were mapped with Bowtie2 (Langmead and Salzberg 2012) to the UCSC Mus musculus mm9 genome, using default settings. Mapped sequence data were analyzed for peaks using HOMER (Hypergeometric Optimization of Motif EnRichment) v4.7 (Heinz et al. 2010). Peak files were annotated to the mouse mm9 genome using HOMER's annotation script.

For manuscripts utilizing custom algorithms or software that are central to the research but not yet described in published literature, software must be made available to editors/reviewers. We strongly encourage code deposition in a community repository (e.g. GitHub). See the Nature Research guidelines for submitting code & software for further information.

Data

Policy information about availability of data

All manuscripts must include a data availability statement. This statement should provide the following information, where applicable:
- Accession codes, unique identifiers, or web links for publicly available datasets
- A list of figures that have associated raw data
- A description of any restrictions on data availability

The sequencing data from this study have been submitted to the NCBI Gene Expression Omnibus (GEO; http://www.ncbi.nlm.nih.gov/geo/) under accession number GSExxxx.

Field-specific reporting

Please select the one below that is the best fit for your research. If you are not sure, read the appropriate sections before making your selection.

☒ Life sciences ☐ Behavioural & social sciences ☐ Ecological, evolutionary & environmental sciences

For a reference copy of the document with all sections, see nature.com/documents/nr-reporting-summary-flat.pdf

Life sciences study design

All studies must disclose on these points even when the disclosure is negative.

Sample size	Data were obtained at least three independent experiments for IF and FC. The amounts of chromatin DNA used for each ChIP-Seq experiment have been reported in Methods.
Data exclusions	No data was excluded from analysis.
Replication	All attempts at replication were successful.
Randomization	B16 cells were randomly seeded on the patterned or non-patterned substrates for further analysis.
Blinding	This study is in vitro experiment and no blinding is required. The investigators were not blinded during data collection and analysis

Reporting for specific materials, systems and methods

We require information from authors about some types of materials, experimental systems and methods used in many studies. Here, indicate whether each material, system or method listed is relevant to your study. If you are not sure if a list item applies to your research, read the appropriate section before selecting a response.

Materials & experimental systems

n/a	Involved in the study	
☐	☒	Antibodies
☐	☒	Eukaryotic cell lines
☒	☐	Palaeontology
☒	☐	Animals and other organisms
☒	☐	Human research participants
☒	☐	Clinical data

Methods

n/a	Involved in the study	
☐	☒	ChIP-seq
☐	☒	Flow cytometry
☒	☐	MRI-based neuroimaging

Antibodies

Antibodies used	CD271 (ABGENT, AM1842a, 1:250 for IF and FC), Jarid1B (BETHYL, A201-813A, 1:500 for IF and FC), Oct4 (ABCAM, AB27985, 1:500 for IF and FC), Sox2 (ABCAM, AB97959, 1:250 for IF and FC), Nanog (SIGMA, N3038, 1:500 for IF and FC), Stat3 (ABCAM, AB119352, 1:500 for IF), a5b1 (MILLIPORE, AAB1969, 1 ug/ml for blocking), H3K4me1 (CELL SIGNALING, 5326, 1:250 for IF and FC), H3K4me2 (CELL SIGNALING, 9725, 1:250 for IF and FC), H3K4me3 (CELL SIGNALING, 9751, 1:250 for IF and FC), H3K36me2 (CELL SIGNALING, 2901, 1:250 for IF and FC), H3K9me3 (CELL SIGNALING, 13969, 1:250 for IF and FC), HDAC1 (CELL SIGNALING, 5356, 1:500 for IF and FC), HDAC2 (CELL SIGNALING, 5113, 1:500 for IF and FC), HDAC3 (CELL SIGNALING, 3949, 1:500 for IF and FC), Ack (CELL SIGNALING, 9441, 1:500 for IF and FC), H3K4ac (ABCAM, AB113672, 1:250 for IF and FC), H3K9ac (CELL SIGNALING, 9649, 1:250 for IF and FC & 1:1000 for WB), PRDM14 (ABClonal, A13658, 1:250 for IF & 1:1000 for WB), and SOX10 (ABCAM, AB212843, 1:500 for IF).<br>Among them H3K4me2 (CELL SIGNALING, 9725) & H3K9ac (CELL SIGNALING, 9649) & SOX10 (ABCAM, AB212843) were used for ChiP-seq.
Validation	All the antibodies above were with reactivity for Human and Mouse and confirmed for IF from each vendor. These were used according to the manufacturer's website.<br>H3K4me2 (CELL SIGNALING, 9725) & H3K9ac (CELL SIGNALING, 9649) were confirmed for ChIP application from CELL SIGNALING.<br>H3K4me2: https://www.cellsignal.com/products/primary-antibodies/di-methyl-histone-h3-lys4-c64g9-rabbit-mab/9725<br>H3K9ac: https://www.cellsignal.com/products/primary-antibodies/acetyl-histone-h3-lys9-c5b11-rabbit-mab/9649<br>SOX10: https://www.abcam.com/sox10-antibody-sox10991-bsa-and-azide-free-ab212843.html

Eukaryotic cell lines

Policy information about cell lines

Cell line source(s)	B16F0 murine melanoma cells from ATCC (ATCC® CRL-6322™).
Authentication	Cells were authenticated from ATCC.
Mycoplasma contamination	The cell line was tested for mycoplasma contamination. Cells were not contaminated.
Commonly misidentified lines (See ICLAC register)	No commonly misidentified cell lines were used.

ChIP-seq

Data deposition

☒ Confirm that both raw and final processed data have been deposited in a public database such as GEO.

☒ Confirm that you have deposited or provided access to graph files (e.g. BED files) for the called peaks.

Data access links *May remain private before publication.*	The sequencing data are available for review at the GEO https://www.ncbi.nlm.nih.gov/geo/query/acc.cgi?acc=GSE146444.
Files in database submission	P H3K9ac_ChIPSeq (GSM4385551), NP H3K9ac_ChIPSeq (GSM4385552), P H3K4me2_ChIPSeq (GSM4385553), NP H3K4me2_ChIPSeq (GSM4385554), P H3Input_ChipSeq (GSM4385555), NP H3Input_ChipSeq (GSM4385556), P Sox10_ChIPSeq (GSM4385557), NP Sox10_ChIPSeq (GSM4385558), P Sox10_Input (GSM4385559), NP Sox10_Input (GSM4385560)
Genome browser session (e.g. UCSC)	We have deposited XLSX files at the GEO.

Methodology

Replicates	Three replicates of each stage-specific H3K4me2, H3K9ac, Sox10-ChIP-Seq were performed and two replicates were performed for stage-specific H3K9ac-ChIP-Seq.
Sequencing depth	Libraries were size selected using AmpureXP beads (Beckman Coulter, Brea, CA, USA), with protocol as written, selecting for DNA between 200-500bp in size. All samples were sequenced in single end format with fragment length of 100 bp.
Antibodies	Described above.
Peak calling parameters	Samples were converted into tag directories, and QC was performed using read mapping and GC bias statistics. Histone peaks were then called from the Tag Directories with default factor settings, except local filtering was disabled (-L 0) and input filtering was set at three-fold over background (-F 3), to increase the sensitivity of the peak calling and identify individual subunits of multihistone peaks. After peak calling, peak files were annotated to the mouse mm9 genome using HOMER's annotation script to assign peaks to genes, and associate peaks with differential expression data.<br>P_k4me2_pool Tag Count in given bp (55983451.0 Total, normalization factor = 0.18, effective total = 10000000)<br>NP_k4me2_pool Tag Count in given bp (47824175.0 Total, normalization factor = 0.21, effective total = 10000000)<br>P_k9ac_pool Tag Count in given bp (54066510.0 Total, normalization factor = 0.18, effective total = 10000000)<br>NP_k9ac_pool Tag Count in given bp (53581477.0 Total, normalization factor = 0.19, effective total = 10000000)
Data quality	Differential chromatin peaks were identified using the HOMER getDifferentialPeak.pl script, looking for any peaks that changed at least two-fold between conditions with a significance cutoff of 1 x 10-4. Library quality was checked by Qubit 2.0 and Bioanalyzer (Agilent 2100). The details of data quality including number of called peaks and fold-enrichment for the stage-specific H3K4me3-ChIP-Seq have been described in the submitted files at the GEO https://www.ncbi.nlm.nih.gov/geo/query/acc.cgi?acc=GSE146444.
Software	Sequence data were mapped with Bowtie2 (Langmead and Salzberg 2012) to the UCSC Mus musculus mm9 genome, using default settings. Mapped sequence data were analyzed for peaks using HOMER (Hypergeometric Optimization of Motif EnRichment) v4.7 (Heinz et al. 2010). BigWiggle pileup files were generated using HOMER's makeBigWig.pl script with default settings. Differential chromatin peaks were identified using the HOMER getDifferentialPeak.pl script. Genes annotated nearby differential peaks were submitted for GO analysis to DAVID and GREAT. Motif Analysis was performed with the HOMER findMotifsGenome.pl script using default settings.

Flow Cytometry

Plots

Confirm that:

☒ The axis labels state the marker and fluorochrome used (e.g. CD4-FITC).

☒ The axis scales are clearly visible. Include numbers along axes only for bottom left plot of group (a 'group' is an analysis of identical markers).

☒ All plots are contour plots with outliers or pseudocolor plots.

☒ A numerical value for number of cells or percentage (with statistics) is provided.

Methodology

Sample preparation	After culturing cells on patterned or non-patterned substrates, they were detached from the substrates, followed by breaking down into a single cell suspension. Cells were fixed in 4% paraformaldehyde for 20 min and then permeabilized in 0.1% Triton X-100 in PBS for 30 min. After blocking cells in 1% BSA in PBS for 1 h, Cells were stained with primary antibodies in 1% BSA in PBS overnight at 4°C and then secondary antibodies in 2% goat serum, 1% BSA in PBS for 20 min in a humid chamber (5% CO_2 and 37°C). Before every step, cells were rinsed at least three times with PBS.
Instrument	A BD LSR Fortessa Flow Cytometry Analyzer was used to perform flow cytometry analysis.
Software	DeNovo Software's FCS Express version 6
Cell population abundance	B16F0 murine melanoma cells
Gating strategy	To set the baseline, negative controls were prepared by staining cells without primary antibodies.

☒ Tick this box to confirm that a figure exemplifying the gating strategy is provided in the Supplementary Information.
