# Supplementary material for: Geometric regulation of histone state directs melanoma reprogramming
Source: Commun Biol. 2020 Jul 3;3:341. doi: 10.1038/s42003-020-1067-1 (PMC7334222; doi:10.1038/s42003-020-1067-1)
Supplement: Supplementary file 2 — Description of Additional Supplementary Files [file 42003_2020_1067_MOESM2_ESM.pdf]

Description of Additional Supplementary Files

File Name: Supplementary Data 1
Description:
Tab 1. The source data underlying the graphs presented in Fig. 1c.
Tab 2. The source data underlying the graphs presented in Fig. 2c and e.
Tab 3. The source data underlying the graphs presented in Fig. 4a, b, c, d, e.
Tab 4. The source data underlying the graphs presented in Supplementary Fig. 1c.
Tab 5. The source data underlying the graphs presented in Supplementary Fig. 2b, c, d.
Tab 6. The source data underlying the graphs presented in Supplementary Fig. 3.
Tab 7. The source data underlying the graphs presented in Supplementary Fig. 4b, c, d, e.
Tab 8. The source data underlying the graphs presented in Supplementary Fig. 5.
Tab 9. The source data underlying the graphs presented in Supplementary Fig. 7b, c, d, e, g, h, i.
Tab 10. The source data underlying the graphs presented in Supplementary Fig. 10.
Tab 11. The source data underlying the graphs presented in Supplementary Fig. 13a, b.
