# Supplementary material for: Geometric regulation of histone state directs melanoma reprogramming
Source: Commun Biol. 2020 Jul 3;3:341. doi: 10.1038/s42003-020-1067-1 (PMC7334222; doi:10.1038/s42003-020-1067-1)
Supplement: Supplementary file 4 — Supplementary Information [file 42003_2020_1067_MOESM4_ESM.pdf]

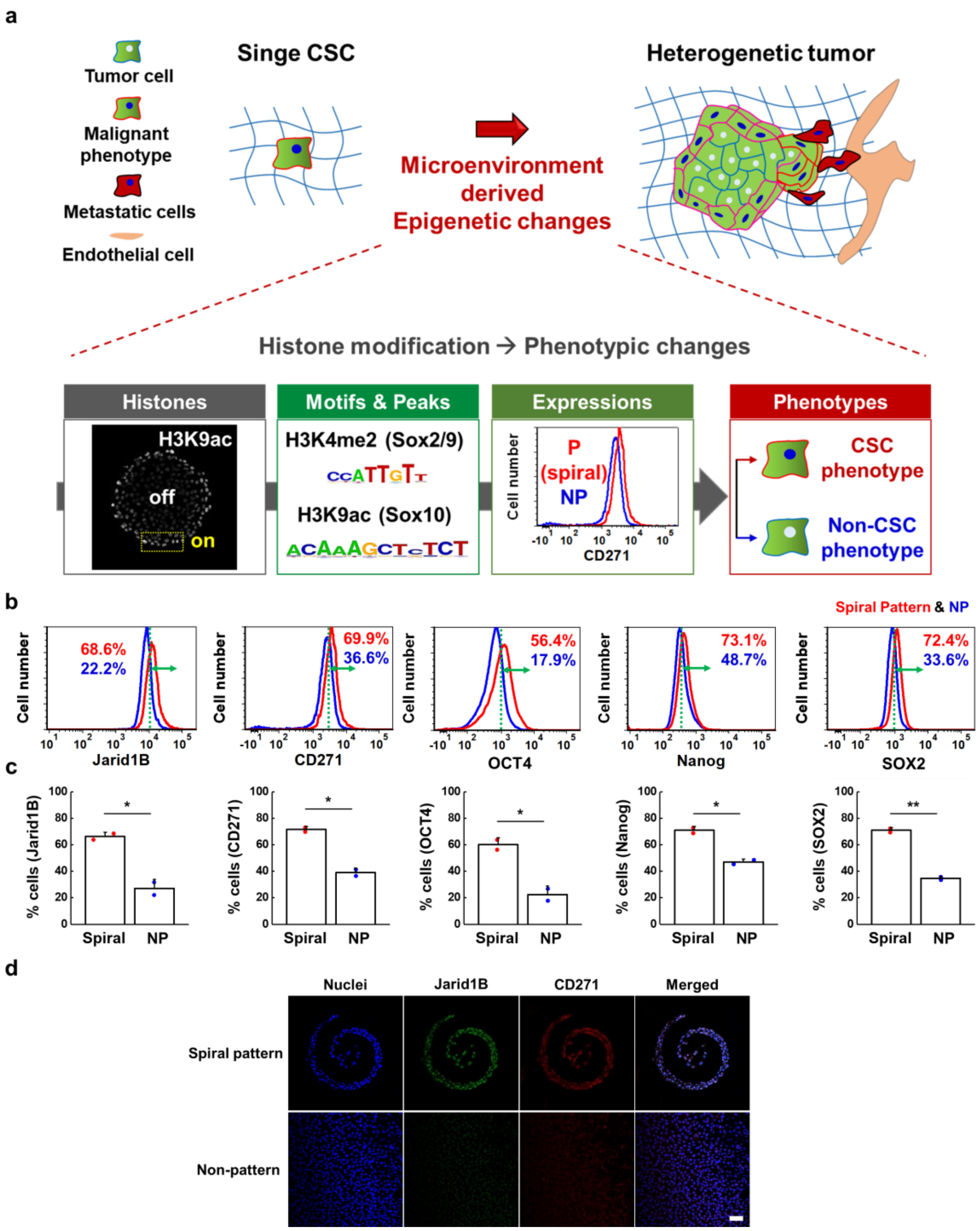

Supplementary Fig. 1. a Scheme of phenotypic alterations caused by histone modifications in response to microenvironment derived epigenetic changes. **b** Flow cytometry characterization of MIC (Jarid1B and CD271) and stemness (Oct4, Nanog, and Sox2) markers in B16F0 cells cultured on spiral patterned or non-patterned substrates. **c** Quantification of cell ration above the thresholds (n = 2, biological replicates). **d** Representative confocal images of Jarid1B and CD271 for B16F0 cells cultured on spiral patterned or non-patterned substrates. Scale bars, 50 µm. *$P < 0.05$, **$P < 0.005$, ANOVA. Error bars represent s.d.

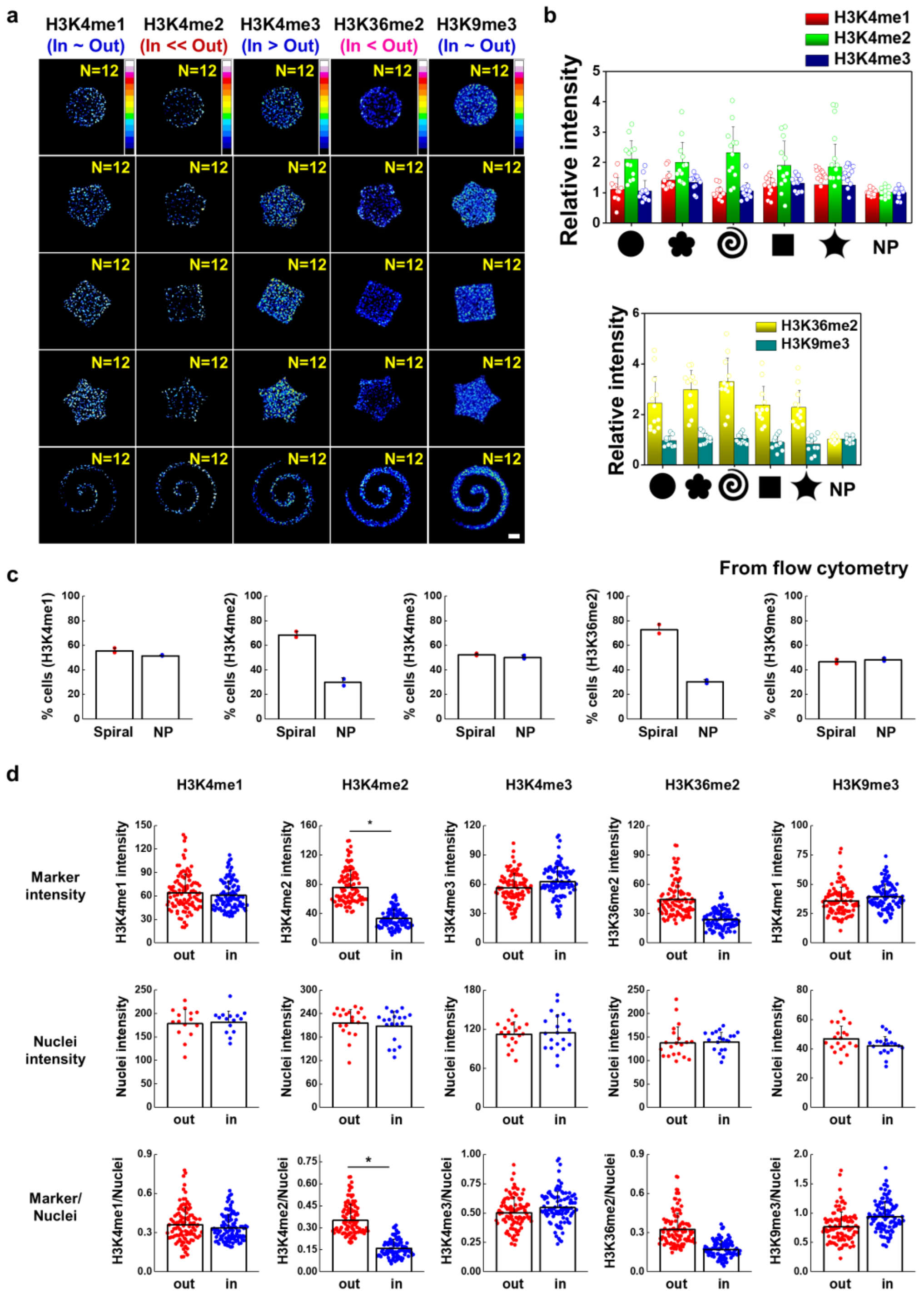

Supplementary Fig. 2. **a** Immunofluorescence heatmaps of methylation (H3K4me3/2/1, H3K36me2, and H3K9me3) for B16F0 cells cultured in a panel of shapes. **b** Histone expression of methylation markers for B16F0 cells cultured in a panel of shapes or non-patterned substrates (n = 3, biological replicates, total 12 patterns for each condition). **c** Quantification of cell ration above the thresholds from the flow cytometry analysis (n = 2, biological replicates). **d** Intensity of methylation (H3K4me3/2/1, H3K36me2, and H3K9me3) and nuclei for B16F0 cells cultured in the circular shapes. Intensity ratio of histone methylation markers normalized by the fluorescent signal to the intensity of DAPI in B16F0 cells cultured in perimeter or central regions of the circular geometry (n = 3, biological replicates, total 100 cells for each condition). Scale bars, 50 μm. *P < 0.05, ANOVA. Error bars represent s.d.

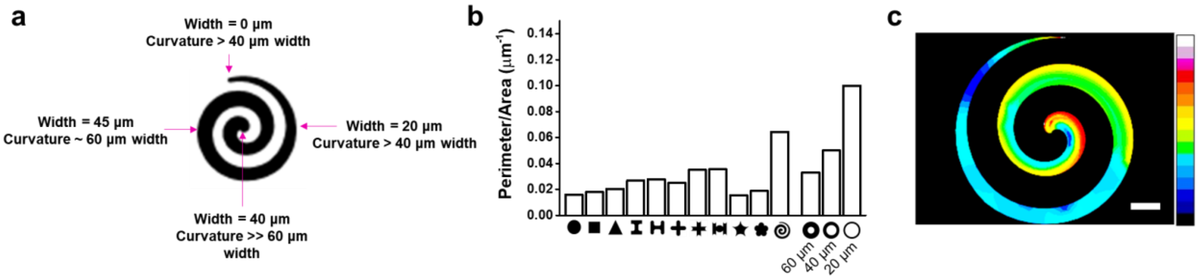

Supplementary Fig. 3. **a** Width and curvature along the spiral shape. **b** Comparison of Perimeter/Area for various geometries. **c** Finite element model of perimeter stress in the spiral shape. Colour bar indicates maximum (top) to minimum (bottom) intensity. Scale bars, 50 µm.

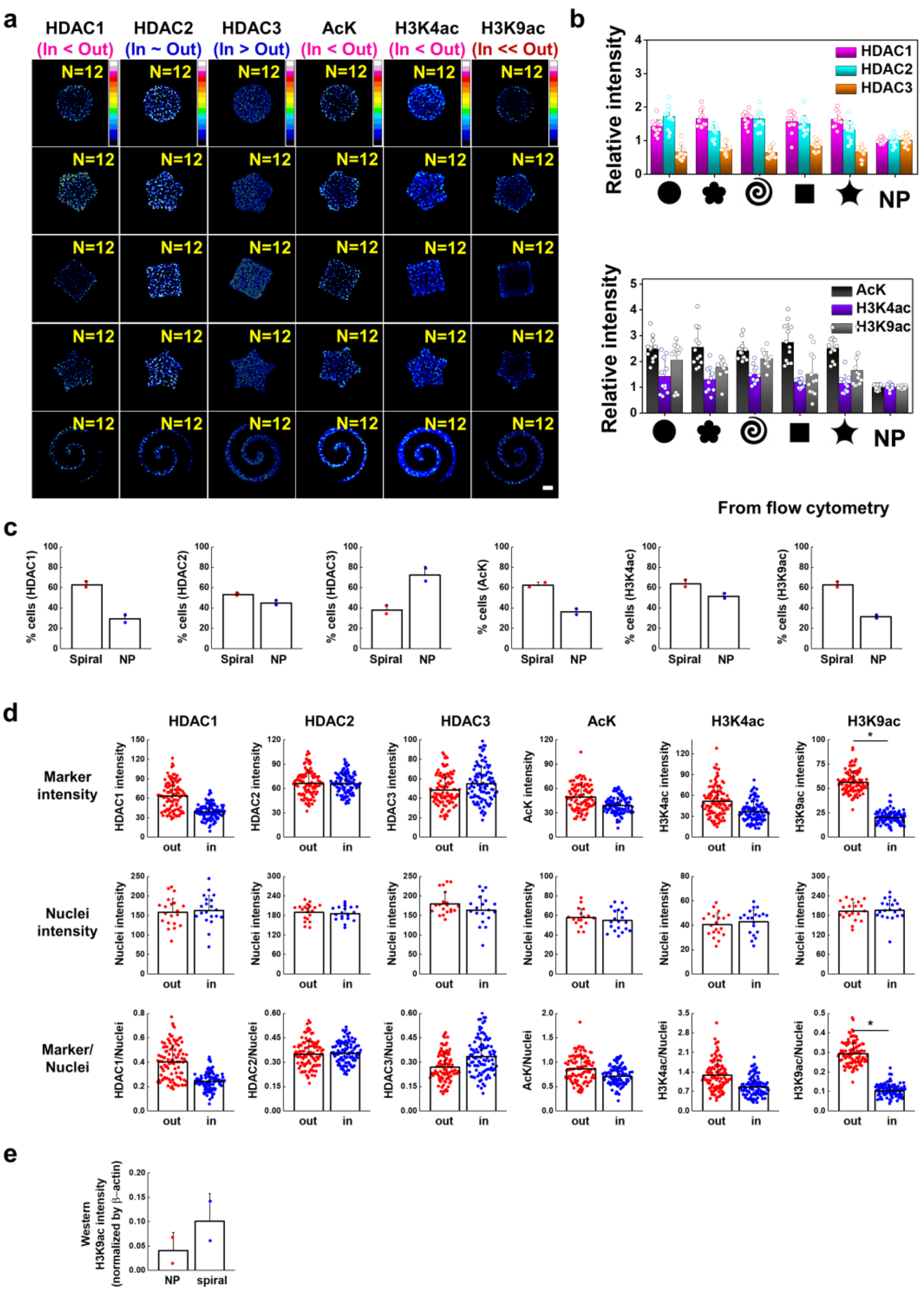

Supplementary Fig. 4. a Immunofluorescence heatmaps of HDAC1/2/3 and acetylation (H3K4ac and H3K9ac) for B16F0 cells cultured in a panel of shapes. **b** Intensity of acetylation markers for B16F0 cells cultured in a panel of shapes or non-patterned substrates (n = 3, biological replicates, total 12 patterns for each condition). **c** Quantification of cell ration above the thresholds from the flow cytometry analysis (n = 2, biological replicates). **d** Intensity of acetylation and deacetylation markers (HDAC1/2/3, AcK, H3K4ac, and H3K9ac) and nuclei for B16F0 cells cultured in the circular shapes. Intensity ratio of histone acetylation and deacetylation markers normalized by the fluorescent signal to the intensity of DAPI in B16F0 cells cultured in perimeter or central regions of the circular geometry (n = 3, biological replicates, total 100 cells for each condition). **e** Quantified difference in H3K9ac relative to the loading control (n = 2, biological replicates). Scale bars, 50 μm. *$P < 0.05$, ANOVA. Error bars represent s.d.

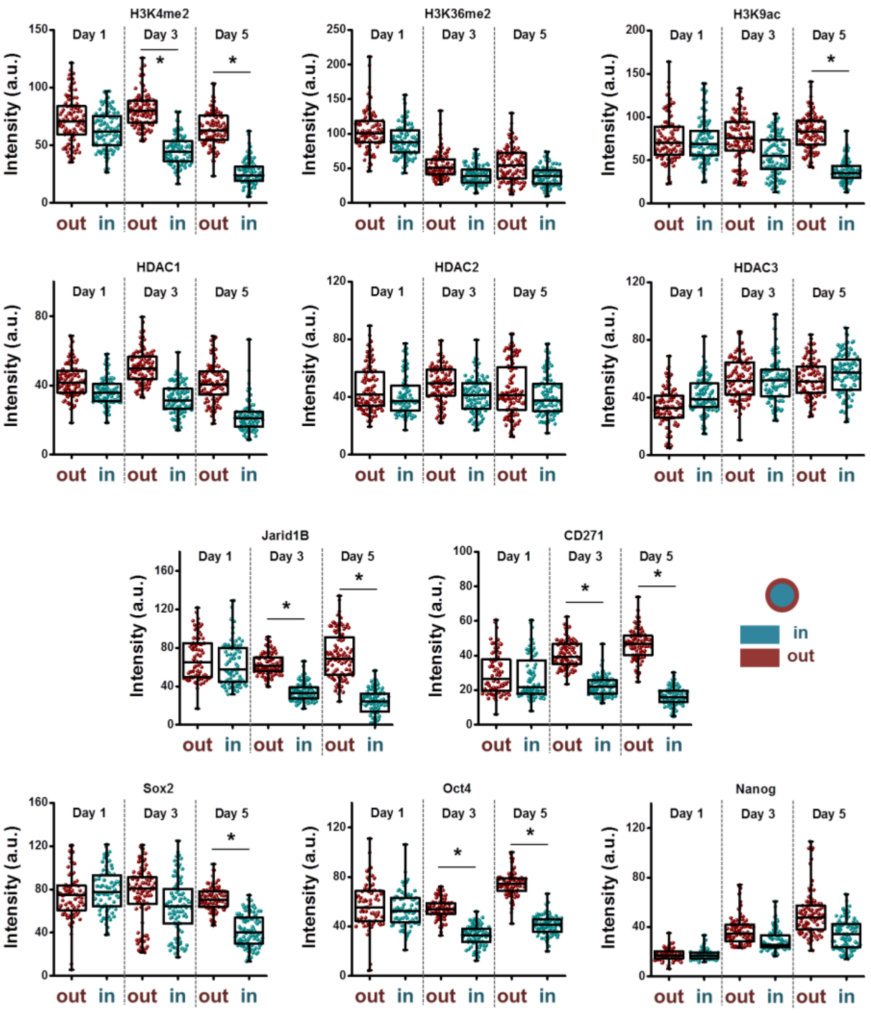

Supplementary Fig. 5. Intensity of Histone modifications (H3K4me2, H3K36me2, H3K9ac, and HDAC1/2/3), MIC markers (Jarid1B and CD271), and transcriptional factors related to stemness and MIC state (Sox2, Oct4, and Nanog) depending on culture time (day 1, 3, and 5) for cells cultured on different regions (outside/inside ratio) of circular shape (n = 3, biological replicates, total 100 cells for each condition except for Jarid1B, CD271, Sox2, Oct4, Nanog on day 1). **P* < 0.05, ANOVA. Error bars represent s.d.

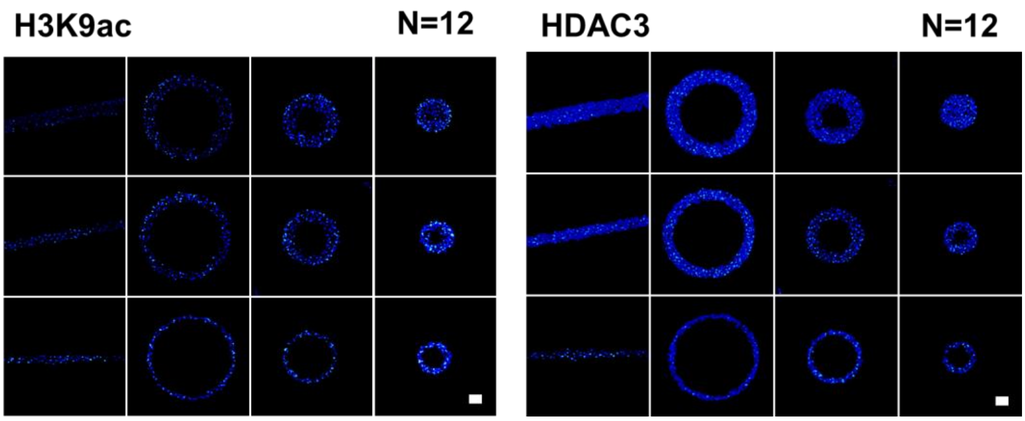

Supplementary Fig. 6. Immunofluorescence heatmaps of H3K9ac and HDAC3 for B16F0 cells cultured in shapes regulating curvature and perimeter/area. Scale bars, 50 μm.

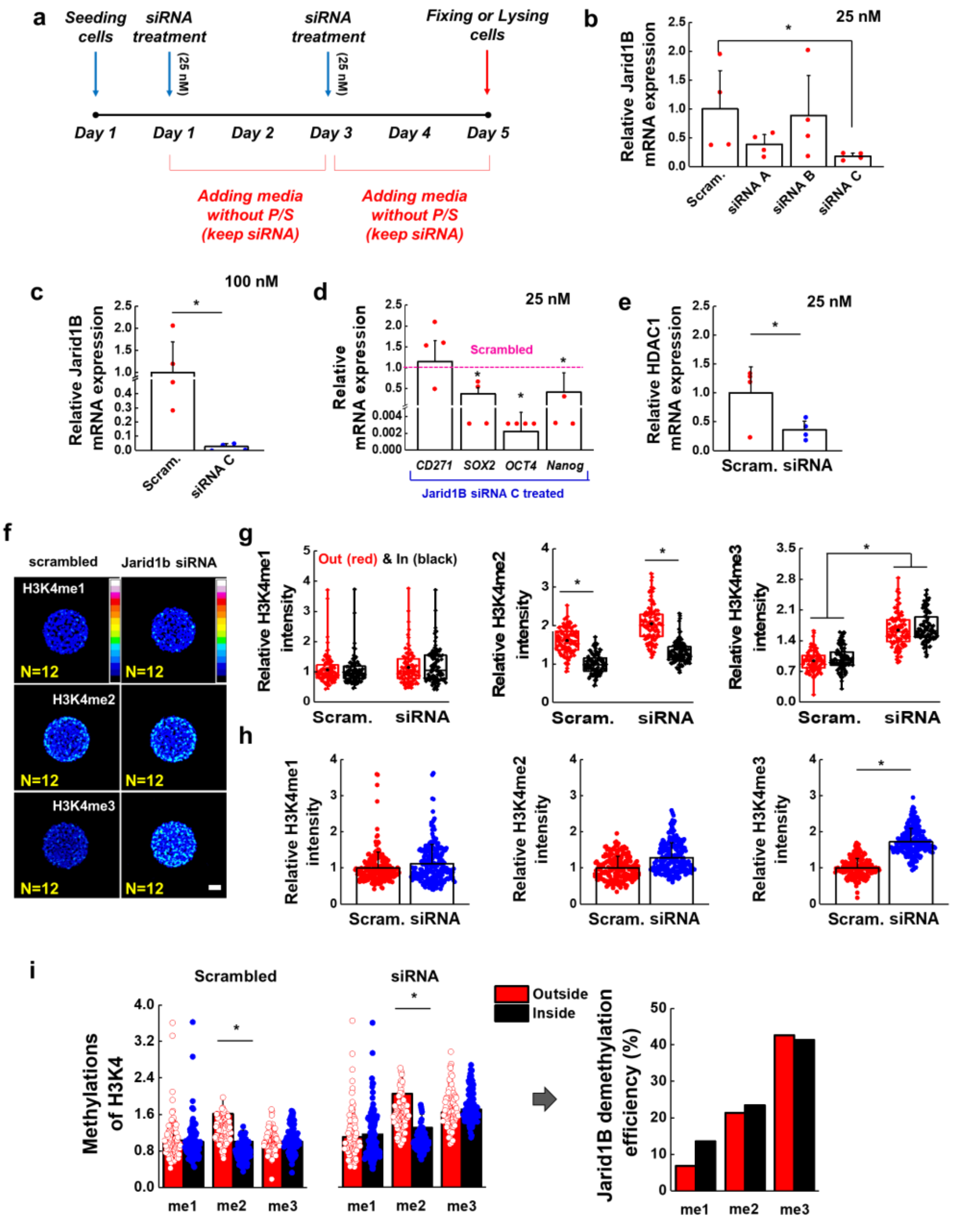

Supplementary Fig. 7. **a** Media conditions for normal, inhibition, or siRNA transfection. Results of real-time PCR to measure the gene expression of Jarid1B (three different sequences of Jarid1B siRNAs (A, B, and C) with different concentrations, **b** 25 or **c** 100 nM. **d** Results of real-time PCR to measure the gene expression of CD271, Sox2, Oct4, and Nanog for B16F0 cells cultured on spiral geometries for 5 days with Jarid1B or scrambled siRNAs and **e** HDAC1 for cells cultured on spiral geometry for 5 days with Jarid1B or scrambled siRNAs (n = 4, biological replicates, except for *Nanog*: n = 3, biological replicates). **f** Immunofluorescence heatmaps of H3K4me3/2/1expression for B16F0 cells cultured on circular geometries treated with scrambled or Jarid1B siRNA. **g** Jarid1B regulates the levels of demethylation of H3K4me3/2/1 with different efficiencies (n = 4, biological replicates, total 100 cells fpr each condition). Intensity values were normalized by the value from cells cultured in the inner side with scrambled. **h** Quantification of general differences in H3K4me1/2/3 levels. Itensity values were normalized by scrambled. **i** Histone H3K4me3/2/1 expression for B16F0 cells cultured in perimeter or central regions of the circular geometry (n = 4, biological replicates), and calculated Jarid1B demethylation efficiency through the composition between cells cultured with scrambled and Jarid1B siRNA. *$P < 0.05$, ANOVA. Error bars represent s.d.

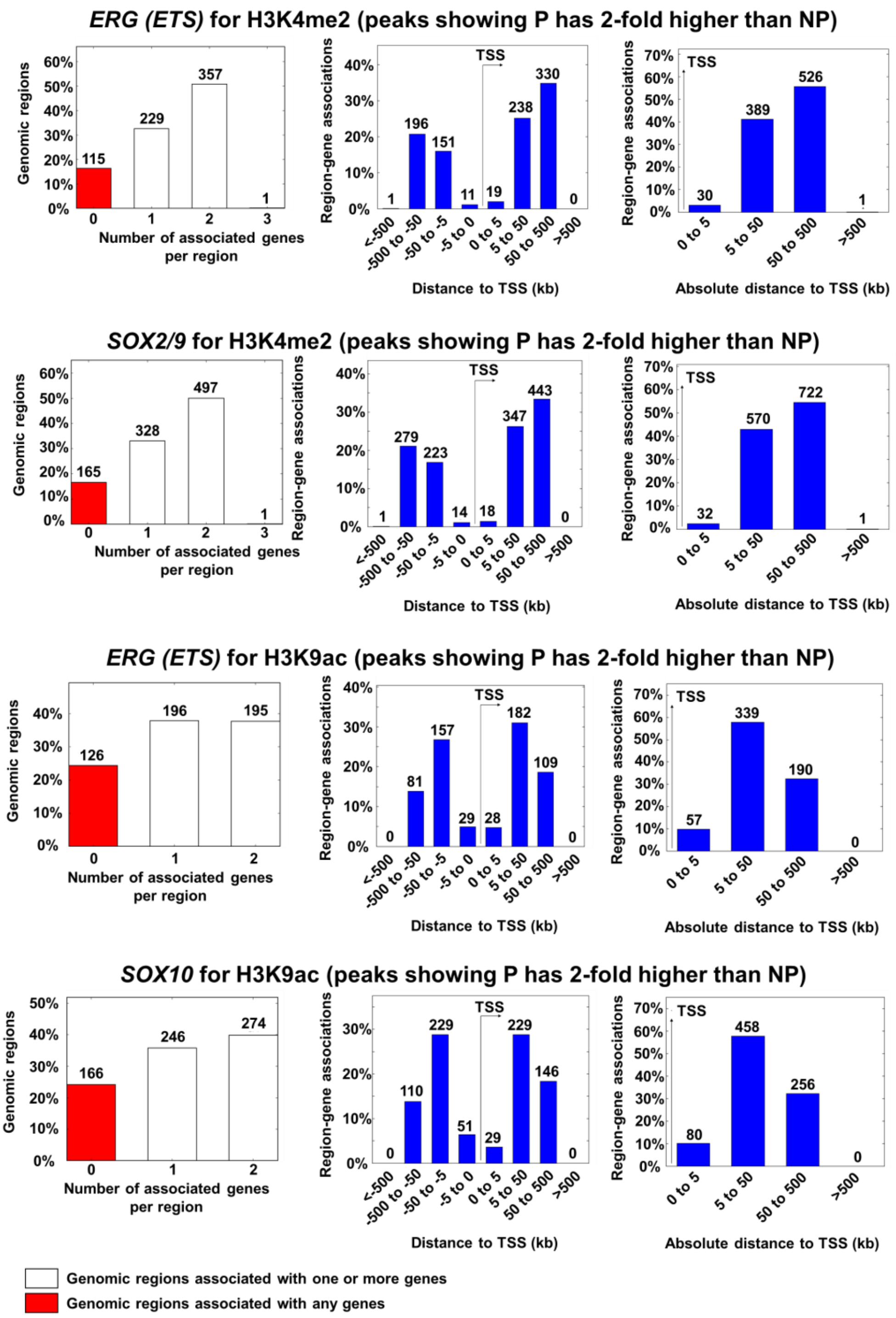

Supplementary Fig. 8. Number of associated genes per region and binned by distance (with orientation or absolute value) to generate enriched annotations (GREAT) of genes for cells cultured on spiral patterns that contain a specific motif (*SOX* or *ETS* family) within the promoter.

a

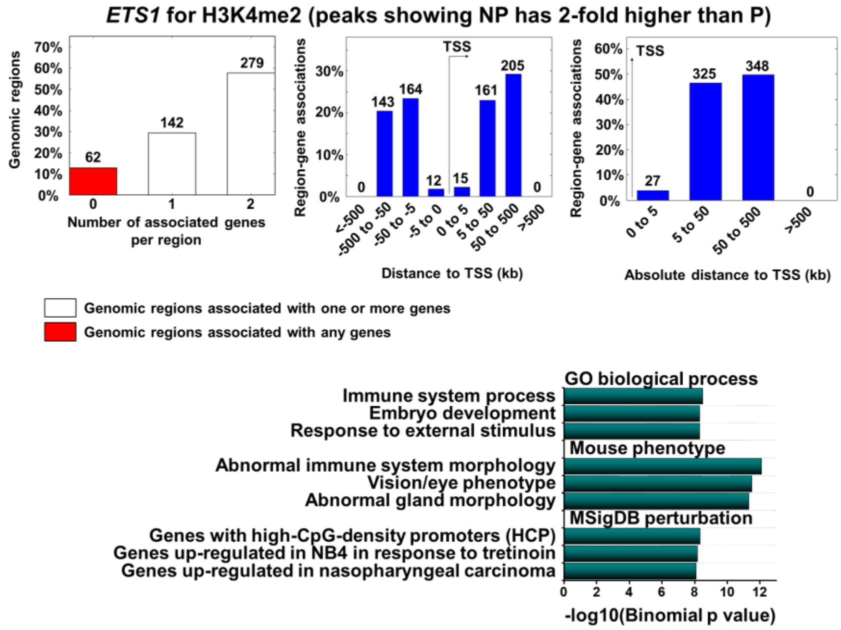

b

Rank	Motif	P-value	log P-pvalue	% of Targets	% of Background	STD(Bg STD)	Best Match/Details	Motif File
1	CTTCCTGT	1e-21	-4.917e+01	36.11%	25.53%	55.9bp (60.2bp)	ERG(ETS)/VCaP-ERG-ChIP-Seq(GSE14097)/Homer(0.948) More Information | Similar Motifs Found	motif file (matrix)
2	ACAAAGCTCTCT	1e-19	-4.483e+01	2.36%	0.36%	47.6bp (55.8bp)	MA0442.1_SOX10/Jaspar(0.667) More Information | Similar Motifs Found	motif file (matrix)
3	AGTCATGTGACT	1e-16	-3.802e+01	2.78%	0.60%	61.7bp (55.0bp)	MITF(bHLH)/MastCells-MITF-ChIP-Seq(GSE48085)/Homer(0.855) More Information | Similar Motifs Found	motif file (matrix)
4	ACAAAAGGCCGGATCT	1e-16	-3.741e+01	0.47%	0.00%	54.0bp (0.0bp)	PB0020.1_Gabpa_1/Jaspar(0.576) More Information | Similar Motifs Found	motif file (matrix)
5	GGGGGTCTCCCTGAG	1e-15	-3.536e+01	0.89%	0.04%	45.6bp (59.1bp)	MF0003.1_REL_class/Jaspar(0.580) More Information | Similar Motifs Found	motif file (matrix)
6	AGTGGTCTCATG	1e-14	-3.313e+01	0.53%	0.01%	46.0bp (35.3bp)	PB0196.1_Zbtb7b_2/Jaspar(0.662) More Information | Similar Motifs Found	motif file (matrix)
7	CTTCTTTTGACA	1e-14	-3.313e+01	0.53%	0.01%	38.6bp (50.2bp)	NF1:FOXA1(CTF,Forkhead)/LNCAP-FOXA1-ChIP-Seq(GSE27824)/Homer(0.610) More Information | Similar Motifs Found	motif file (matrix)
8	GACTTCTTTG	1e-14	-3.240e+01	0.95%	0.06%	48.5bp (59.5bp)	PRDM1(Zf)/Hela-PRDM1-ChIP-Seq(GSE31477)/Homer(0.609) More Information | Similar Motifs Found	motif file (matrix)
9	TTCCTGATTAGTGTG	1e-13	-3.198e+01	0.41%	0.00%	51.7bp (19.9bp)	Ets1-distal(ETS)/CD4+-PolII-ChIP-Seq(Barski et al.)/Homer(0.577) More Information | Similar Motifs Found	motif file (matrix)
10	ACTTAATT	1e-13	-3.103e+01	26.24%	18.78%	56.2bp (60.3bp)	MA0075.1_Prrx2/Jaspar(0.795) More Information | Similar Motifs Found	motif file (matrix)
11	AAGCACAGTGTCTT	1e-13	-2.998e+01	1.42%	0.19%	59.6bp (60.8bp)	RUNX(Runt)/HPC7-Runx1-ChIP-Seq(GSE22178)/Homer(0.549) More Information | Similar Motifs Found	motif file (matrix)
12	GGGTGAATAA	1e-12	-2.974e+01	1.18%	0.12%	51.9bp (65.9bp)	PB0013.1_Eomes_1/Jaspar(0.688) More Information | Similar Motifs Found	motif file (matrix)
13	GAGTCAAA	1e-12	-2.880e+01	14.18%	8.81%	55.0bp (59.7bp)	MA0099.2_JUN::FOS/Jaspar(0.793) More Information | Similar Motifs Found	motif file (matrix)
14	ATTCAACACCAGGTA	1e-12	-2.868e+01	0.47%	0.01%	35.7bp (42.0bp)	MA0138.2_REST/Jaspar(0.545) More Information | Similar Motifs Found	motif file (matrix)
15 *	TTCCCTGAGCAGGAG	1e-11	-2.716e+01	0.41%	0.01%	64.1bp (1.2bp)	Unknown-ESC-element(?)/mES-Nanog-ChIP-Seq(GSE11724)/Homer(0.593) More Information | Similar Motifs Found	motif file (matrix)
16 *	ATGATAACAGAC	1e-11	-2.698e+01	0.53%	0.01%	62.2bp (52.9bp)	PB0119.1_Foxa2_2/Jaspar(0.675) More Information | Similar Motifs Found	motif file (matrix)

Supplementary Fig. 9. a Number of associated genes per region and binned by distance (with orientation or absolute value) to generate enriched annotations (GREAT) of genes for cells cultured on non-patterned substrates that contain a specific motif (*ETS1* family) within the promoter, and the enriched annotation results. **b** The top 16 predictive transcription factor motifs (H3K9ac, P vs. NP) with p-values.

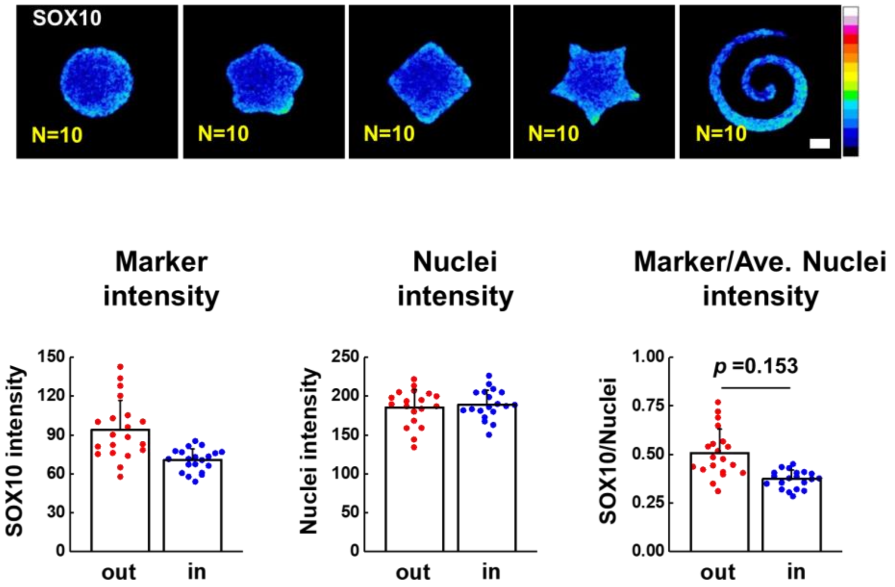

Supplementary Fig. 10. Immunofluorescence heatmaps of SOX10 for B16F0 cells cultured in a panel of shapes. Intensity ratio of SOX10 normalized by the fluorescent signal to the intensity of DAPI (n = 3, biological replicates, total 20 patterns for each condition. Scale bars, 50 µm. *P* value from ANOVA. Error bars represent s.d.

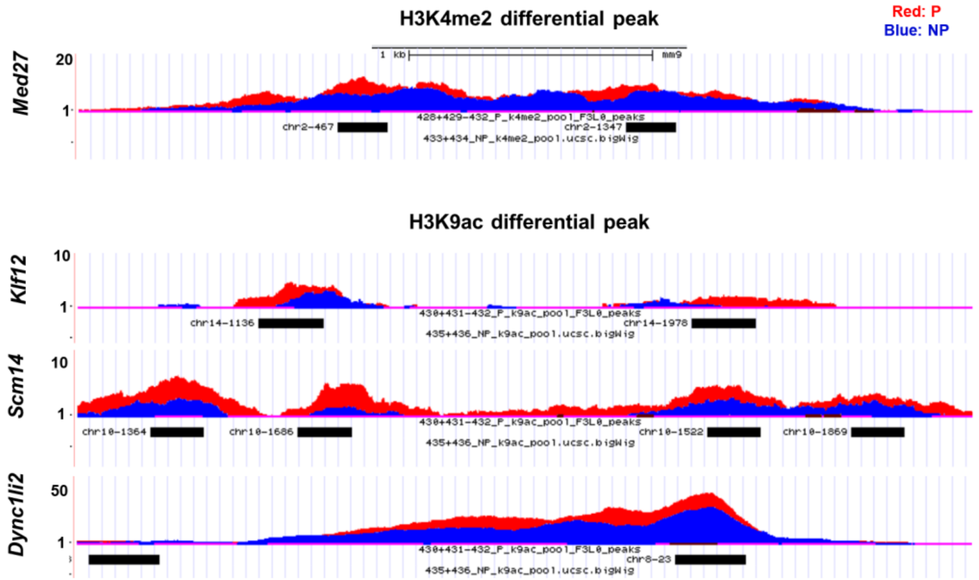

Supplementary Fig. 11. Differential peaks of H3K4me2 (*Med27* and *Trim14*) and H3K9ac (*Klf12*, *Scml4*, and *Dync1li2*) associated with cells cultured on patterned gels compared to those cultured on non-patterned gels.

a

GENE NAME
EP300 interacting inhibitor of differentiation 2(Eid2)
Iroquois related homeobox 6 (Drosophila)(Irx6)
JAZF zinc finger 1(Jazf1)
Kruppel-like factor 12(Klf12)
MyoD family inhibitor domain containing(Mdfic)
T-box 4(Tbx4)
TOX high mobility group box family member 3(Tox3)
engrailed 1(En1)
histone deacetylase 7(Hdac7)
low density lipoprotein receptor-related protein 6(Lrp6)
sex comb on midleg-like 4 (Drosophila)(Scml4)
zinc finger protein 600(Zfp600)
zinc finger protein 982(Zfp982)
zinc finger protein 985(Zfp985)

b

GENE NAME
EP300 interacting inhibitor of differentiation 2(Eid2)
T-box 4(Tbx4)
engrailed 1(En1)
low density lipoprotein receptor-related protein 6(Lrp6)
sema domain, immunoglobulin domain (Ig), transmembrane domain (TM) and short cytoplasmic domain, (semaphorin) 4D(Sema4d)

Supplementary Fig. 12. a The top enriched GO category regulating transcription. **b** The other TFs that *SOX10* binds nearby in B16s.

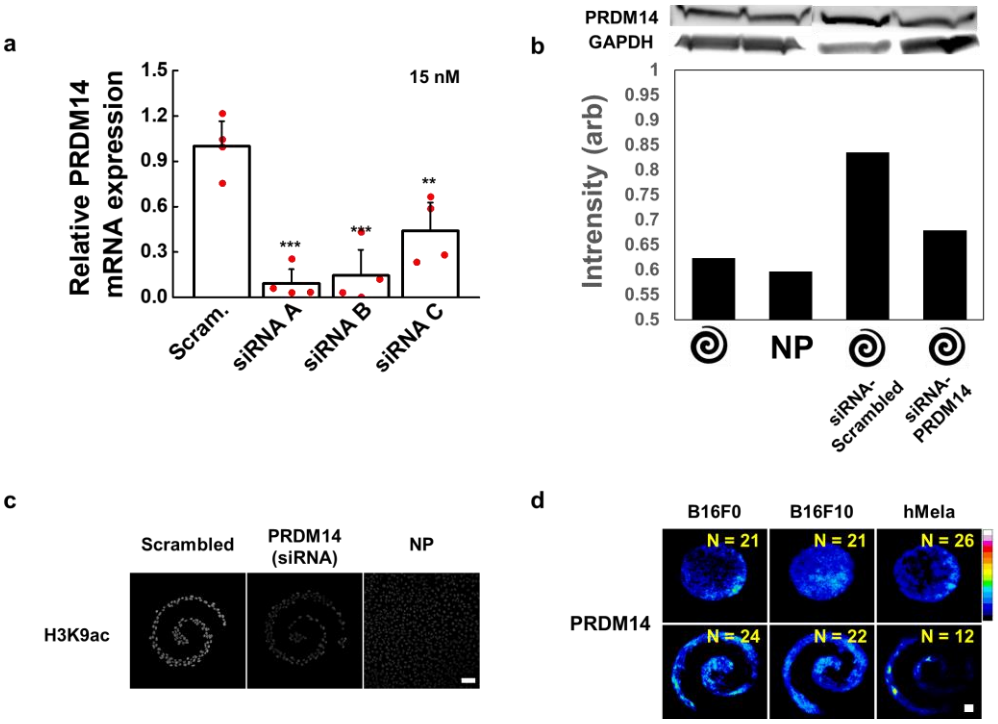

Supplementary Fig. 13. a Results of real-time PCR to measure the gene expression of *PRDM14* (three different sequences of *PRDM14* siRNAs (A, B, and C) with 15 nM concentrations. **b** Western blots for *PRDM14* with scrambled or siRNA against *PRDM14*. **c** Representative immunofluorescence images of H3K9ac for B16F0 cells cultured in the spiral shapes with scrambled or *PRDM14* siRNAs. A representative image of H3K9ac for B16F0 cells cultured on non-patterned substrates to compare the level of H3K9ac to patterned cells with *PRDM14* siRNAs. **d** Immunofluorescence heatmaps of PRDM14 for B16F0, B16F10, and hMela cells cultured in circular and sprial shapes. (Scale bars, 50 µm. $^{**}P < 0.005$, $^{***}P < 0.0005$, ANOVA. Error bars represent s.d.

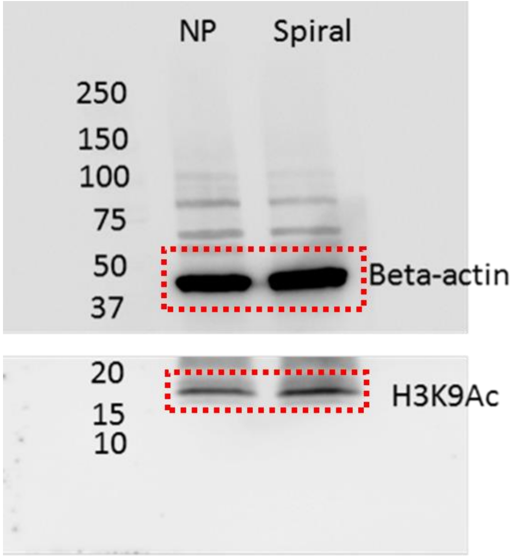

Supplementary Fig. 14. Uncropped western blot image corresponding to Fig. 2d.

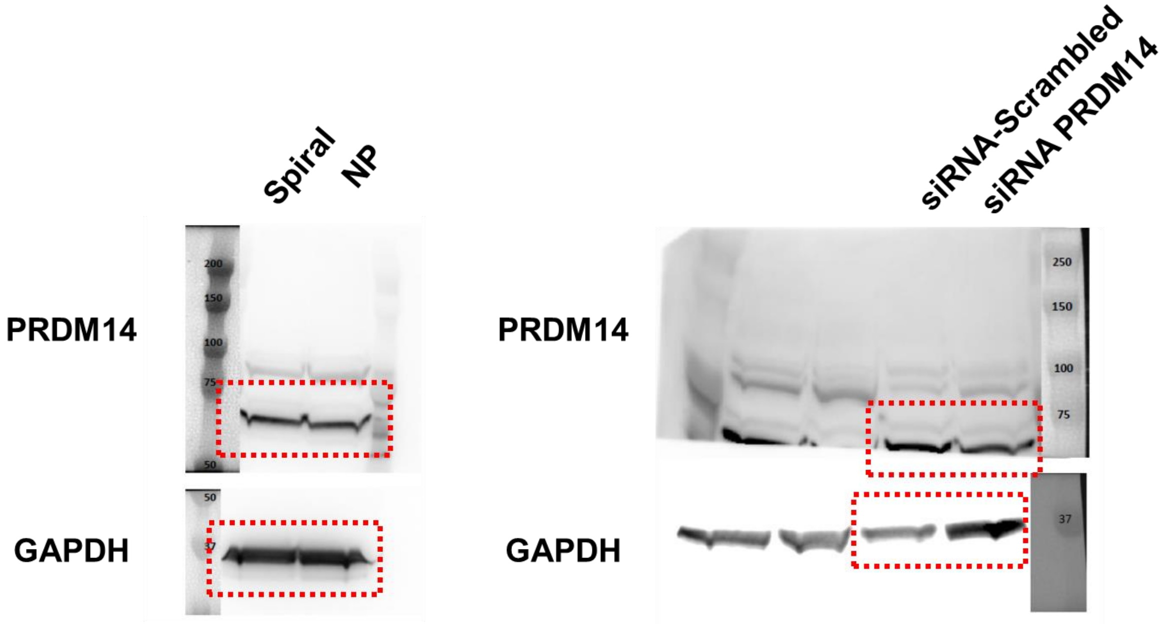

Supplementary Fig. 15. Uncropped western blot image corresponding to Supplementary Fig. 13b.

Sample	# overlaps	p-value
P_k4me2	1758	2.74E-28
P_k9ac	870	9.72E-22
NP_k4me2	1652	2.14E-25
NP_k9ac	917	5.18E-23
PvsNP_k4me2	42	4.13E-01
PvsNP_k9ac	52	2.21E-02
NPvsP_k4me2	40	2.28E-02
NPvsP_k9ac	61	1.06E-03

Supplementary Table 1. Peak co-occurrence of *PRDM14* with the H3K9ac and H3K4me2 marks from our study within 1000bp.

Name	Company	Catalog #
High Glucose DMEM	Corning	10-013-CV
Fetal bovine serum (FBS)	Denville	FB5001
12 well plates	VWR	10062-894
pen streptomycin	GIBCO	15140122
Trypsin 0.25%	GIBCO	15050-065
Sodium periodate	SIGMA-ALDRICH	311448
Fibronectin	SIGMA	F2006

Supplementary Table 2. Reagent information for cell culture.

Antibody	Company	Catalog #	Dilution/ Application
DAPI	INVITROGEN	D3571	1:5000/IF
Actin	INVITROGEN	A12379	1:200/IF
Goat 488-anti-rabbit	ABCAM	AB150077	1:200/IF
Goat 647-anti-mouse	ABCAM	AB150115	1:200/IF
CD271	ABGENT	AM1842a	1:250/IF, FC
Jarid1B	BETHYL	A301-813A	1:500/IF, FC
Oct4	ABCAM	AB27985	1:500/IF, FC
Sox2	ABCAM	AB97959	1:250/IF, FC
Nanog	SIGMA	N3038	1:500/IF, FC
Stat3	ABCAM	AB119352	1:500/IF
a5b1	MILLIPORE	MAB1969	1 µg/ml, blocking
H3K4me1	CELL SIGNALING	5326	1:250/IF, FC
H3K4me2	CELL SIGNALING	9725	1:250/IF, FC
H3K4me3	CELL SIGNALING	9751	1:250/IF, FC
H3K36me2	CELL SIGNALING	2901	1:250/IF, FC
H3K9me3	CELL SIGNALING	13969	1:250/IF, FC
HDAC1	CELL SIGNALING	5356	1:500/IF, FC
HDAC2	CELL SIGNALING	5113	1:500/IF, FC
HDAC3	CELL SIGNALING	3949	1:500/IF, FC
AcK	CELL SIGNALING	9441	1:500/IF, FC
H3K4ac	ABCAM	AB113672	1:250/IF, FC
H3K9ac	CELL SIGNALING	9649	1:250/IF, FC
PRDM14	ABCLONAL	A13658	1:500/IF
SOX10	ABCAM	AB212843	1:500/IF

Supplementary Table 3. Antibody information for immunostaining, flow cytometry analysis, and integrin blocking.

Primer	Forward	Reverse
Jarid1B	GACATCACAAGCGAATGGTG	CGCTTTCATCCACAAGATCC
CD271	GGG GGT AGA CCT TGT GAT CC	GTG TGC GAG GAC ACT GAG C
OCT4	TGC CCG AAA CCC ACA CTG	CTC GGA CCA CAT CCT TCT CG
SOX2	TGC TGC CTC TTT AAG ACT AGG AC	CGC CGC CGA TGA TTG TTA TT
Nanog	AAC AGG TGA AGA CCT GGT TCC	GAG GCC TTC TGC GTC ACA C
HDAC1	CAA ATT GTG AGT CAT GCG GA	GGC ACC AAG AGG AAA GTC TG
GAPDH	TGC CTC GAT GGG TGG AGT	GCC CAA TAC GAC CAA ATC AGA

Supplementary Table 4. RT-PCR primer sequence information.
